# Kaempferol attenuates LPS-induced inflammatory responses in H9c2 cells through involvement of the IL-6/JAK2/STAT3 pathway

**DOI:** 10.1007/s11033-026-11855-2

**Published:** 2026-04-27

**Authors:** Hongyi Yue, Yunfei Jia, Ruohao Sun, Hehe Liao, Yi Zuo, Pingyi Wang, Wenhua Li

**Affiliations:** 1 Clinical Medical Research Center for Plateau Gastroenterological disease of Xizang Autonomous Region, and School of Medicine, Xizang Minzu University, Xianyang, 712082 China; 2 The Affiliated Hospital of Xizang Minzu University, Shaanxi, 712082 China; 3 Hainan Medical University, Haikou, 570100 China; 4 Department of Rehabilitation Medicine, the Third Affiliated Hospital, Sun Yat-sen University, Guangzhou, 510000 Guangdong Province China; 5 Department of Rehabilitation Medicine, Nuclear Industry 215 Hospital of Shaanxi Province, Xianyang, 712000 Shaanxi Province China; 6 School of Medicine, Xizang Minzu University, Xianyang, 712082 China

**Keywords:** Kaempferol, Lipopolysaccharide, Cytokine storm, Signaling pathway, Oxidative stress

## Abstract

**Background:**

Kaempferol (Kae), a flavonoid compound, has shown considerable potential in preventing and treating cardiovascular diseases and ameliorating myocardial damage. However, its underlying mechanisms remain complex and require further elucidation. This study investigated the protective effects of Kae pretreatment against lipopolysaccharide (LPS)-induced inflammatory responses in H9c2 cardiomyocytes through the interleukin-6 (IL-6)/Janus kinase 2 (JAK2)/signal transducer and activator of transcription 3 (STAT3) signaling pathway, aiming to establish a theoretical foundation for Kae development and clinical application.

**Methods:**

Initially, potential targets were identified through network pharmacology and molecular docking. The protective efficacy of Kae against CS was then preliminarily validated in vivo via histopathological analysis (hematoxylin and eosin staining). Finally, an LPS-induced CS model was constructed in H9c2 cardiomyocytes to elucidate the underlying mechanisms in vitro.

**Results:**

Network pharmacology analysis indicated that Kae may exert anti-CS effects via modulation of the IL-6/JAK2/STAT3 signaling pathway. In vivo experiments demonstrated that Kae significantly ameliorated histopathological changes, including disorganized myocardial fibers and inflammatory cell infiltration. Compared with the model group, serum levels of tumor necrosis factor alpha (TNF-α), IL-6, and interleukin-1β (IL-1β) were significantly decreased, along with reduced mRNA expression of these cytokines in cardiac tissue. In vitro, LPS stimulation significantly elevated the levels of TNF-α, IL-6, and IL-1β, and increased phosphorylated Janus kinase 2 (p-JAK2) and phosphorylated signal transducer and activator of transcription 3 (p-STAT3) expression compared with the control group (*P* < 0.01). Kae pretreatment significantly reduced these inflammatory mediators and downregulated p-JAK2 and p-STAT3 expression compared with the LPS group (*P* < 0.01). Additionally, Kae pretreatment increased superoxide dismutase activity while decreasing malondialdehyde content and nitric oxide production (*P* < 0.05).

**Conclusion:**

Kae pretreatment demonstrated a protective effect against the LPS-induced inflammatory response in H9c2 cells. This protective effect potentially involves regulating the IL-6/JAK2/STAT3 signaling pathway.

**Supplementary Information:**

The online version contains supplementary material available at 10.1007/s11033-026-11855-2.

## Introduction

The inflammatory response represents a protective host defense mechanism triggered by harmful stimuli, characterized by the release of multiple inflammatory cytokines such as tumor necrosis factor alpha (TNF-α), interleukin-1β (IL-1β), and interleukin-6 (IL-6) [1]. However, an excessive inflammatory response serves as the pathological basis for the onset and progression of several diseases, including coronavirus disease 2019 (COVID-19) and sepsis. This phenomenon is also referred to as a cytokine storm (CS) [2]. CS can cause increased vascular permeability and acute lung injury, which may progress to acute respiratory distress syndrome (ARDS) and multi-organ failure involving the heart, liver, spleen, and kidneys [3, 4]. In addition, most sepsis patients present with varying degrees of myocardial inflammation. In severe cases, the development of CS is associated with a marked increase in mortality (from approximately 20% to 70%) [5]. Therefore, preventing the occurrence of CS is crucial for halting the progression of the disease. The unclear efficacy and potential adverse effects of the currently available clinical drugs used to inhibit CS significantly limit their widespread use. To effectively prevent the emergence and progression of CS, there is an urgent need to identify new therapeutic agents that demonstrate substantial efficacy.

Kaempferol (Kae) is a dietary flavonoid abundant in plants such as *Camellia sinensis*, cruciferous vegetables, and traditional Chinese herbs (*Kaempferia galanga* L., *Sophora japonica* L., and *Ginkgo biloba* L.) [6]. Because of its wide range of pharmacological effects, including anti-inflammatory, anticancer, and antioxidant properties, Kae has garnered significant attention from scholars in current research [7]. Some studies have found that Kae can enhance endothelial cell function by reducing oxidative stress and inhibiting inflammatory responses, thereby preventing the progression of atherosclerosis [8]. More interestingly, Micek and other scholars found that when the body consumes foods rich in Kae, the risk of cardiovascular disease is significantly reduced, particularly in terms of lowering the risk of stroke and coronary artery disease [9]. Overall, Kae plays a significant role in both anti-inflammatory and cardiovascular protection. However, its specific mechanism of action is complex and requires further investigation.

Our research team previously found in in vivo experiments that the key active component Kae of the Tibetan medicine *Rhodiola grandiflora* can significantly inhibit the surge of inflammatory factors [10]. Based on the results of the preliminary experiment, the first phase of this study employed network pharmacology and molecular docking to explore mechanisms underlying Kae’s anti-CS effects. The findings suggest that the anti-inflammatory effects of Kae may be mediated through the IL-6/JAK2/STAT3 signaling pathway. This hypothesis was further validated through in vivo and vitro experiments, which enhanced our understanding of the mechanism by which Kae exerts its anti-CS effects, thereby providing a new scientific basis for the continued development and application of this drug.

## Materials and methods

### Acquisition of overlapping targets of kae and CS

The chemical structure of Kae was retrieved from the PubChem database. The Comparative Toxicogenomics Database (CTD), GeneCards, PharmMapper, Similarity Ensemble Approach (SEA), Search Tool for Interacting Chemicals (STITCH), and SwissTargetPrediction databases were used to identify protein targets for Kae. Disease-related protein targets were then retrieved from the GeneCards and Online Mendelian Inheritance in Man (OMIM) databases. Data from these sources was integrated, with duplicates removed. The intersection between drug and disease targets was visualized using a Venn diagram. Finally, the Kae-CS target intersection Venn diagram was generated using the Wei Sheng Xin online platform (http://www.bioinformatics.com.cn/).

### Construction of protein interaction networks

To investigate interactions among the intersecting targets, a protein-protein interaction (PPI) network was constructed and analyzed using the STRING database to reveal the molecular mechanisms underlying Kae-mediated CS modulation [11].

### GO annotation and KEGG pathway analysis

The identified targets were subjected to Gene Ontology (GO) and Kyoto Encyclopedia of Genes and Genomes (KEGG) pathway enrichment analysis using the DAVID database [12]. Analysis was restricted to Homo sapiens, with statistical significance determined by enrichment counts and a p-value threshold of < 0.05. The top 40 enriched GO terms and KEGG pathways were subsequently prioritized for detailed analysis.

### Molecular docking of kae with pathway proteins

The 2D structure of Kae was downloaded from PubChem and converted to a 3D model using Chem 3D software. The structure was then energy-minimized to obtain a stable conformation. Using AutoDock Tools 1.5.6, the .mol file was processed by removing water molecules, adding Gasteiger charges, defining atom types, and setting all non-ring bonds as rotatable. The prepared ligand file was subsequently saved in .pdbqt format for docking analysis.

The crystal structures of IL-6, JAK2, and STAT3 (PDB ID: 1ALU, 7F7W, and 6NJS) were retrieved from the PDB database (http://www.rcsb.org). Structure preparation was performed using PyMOL and AutoDock Tools, including removal of water molecules and co-crystallized ligands, addition of polar hydrogens, and assignment of Kollman charges. The processed proteins were saved in .pdbqt format. Molecular docking was conducted with a grid box of 80 × 80 × 80 Å (grid spacing: 1.0 Å) to encompass the binding pocket. Finally, docking poses were visualized using PyMOL.

### Reagents used in the experiment

Kaempferol (Cat# SJ-MN0209) and AG490 (Cat# SJ-MX5315) were purchased from Sparkjade (AG-490, a specific inhibitor of JAK2, suppresses JAK2 activation and consequently reduces STAT3 activation. It is widely used in tumor, immune, and inflammatory research [13]). Lipopolysaccharide (LPS) (Cat# S11060) and Dexamethasone (Dex) (Cat# S17003) were obtained from the Nanjing Jiancheng Bioengineering Institute. CY3 Fluorescent Antibody (Cat# BA1032), β-actin (Cat# BM3873), DAPI Staining Solution (Cat# AR1176), BCA Protein Concentration Assay Kit (Cat# AR0146), DAPI Staining Solution (Cat# AR1176), BCA Protein Concentration Measurement Kit (Cat# AR0146) were purchased from BOSTER. Phospho-STAT3 antibody (Cat# 9145) was purchased from Cell Signaling Technology (Shanghai) Biological Reagents Co., Ltd. Phospho-JAK2 antibody (Cat# ab32101) was obtained from Abcam.

### Animals and treatments

Specific pathogen-free (SPF) male Sprague Dawley rats (aged 6–7 weeks; body weight, 180–200 g) were obtained from Chengdu Dashuo Experimental Animal Co., Ltd. (Sichuan, China). Following a 7-day acclimatization period, the animals were randomly allocated into six experimental groups (*n* = 6 per group): control (Con), lipopolysaccharide (LPS), dexamethasone (Dex), and low-, medium-, and high-dose Kae (Kae-L, Kae-M, and Kae-H) groups.

### Grouping and administration of drugs

#### In vitro experiment

(1) Control (Con) group: H9c2 cells in the control group were cultured in complete DMEM medium without any treatment for 24 h.

(2) Model (LPS) group: H9c2 cells were stimulated with 10 µg/mL LPS for 12 h to establish an inflammatory model (concentration determined based on published literature and preliminary experiments) [14].

(3) Dex group: H9c2 cells were pretreated with 5 µg/mL Dex for 30 min, subsequently exposed to 10 µg/mL LPS, and incubated for a total of 12 h [15].

(4) Different doses of Kae pretreatment group (Kae group): H9c2 cells were pretreated with Kae at varying concentrations (25, 50, and 100 µmol/L; designated as low, medium, and high dose, respectively) for 30 min prior to LPS (10 µg/mL) stimulation. Cells were then co-incubated for 12 h (concentrations selected based on CCK-8 screening and published literature) [16].

(5) AG490 pretreatment group (AG490 group): H9c2 cells were pretreated with AG490 (10 µmol/L) for 30 min, followed by stimulation with LPS (10 µg/mL) and co-incubation for 12 h.

(6) AG490 + Kae high-dose pretreatment group (AG490 + Kae-H group): H9c2 cells were pretreated with AG490 (10 µmol/L) and Kae (100 µmol/L) for 30 min, followed by LPS (10 µg/mL) stimulation and co-incubation for 12 h.

#### In vivo experiment

The doses of LPS, Dex, and Kae used in the in vivo experiments were determined based on relevant literature [17, 18].

1. Con group: For the Con group, rats were pretreated with 0.3 mL sterile saline intraperitoneal (i.p.), then administered 7 mg/kg saline (i.p.) 30 min later.
2. LPS group: For the LPS group, rats were pretreated with 0.3 mL saline (i.p.), then challenged with 7 mg/kg LPS (i.p.) after 30 min.
3. Dex group: For the Dex group, rats were pretreated with 5 mg/kg dexamethasone (i.p.) for 30 min, prior to LPS challenge (7 mg/kg, i.p.).
4. Kae group: For the Kae groups, rats were pretreated with 25 mg/kg (Kae-L), 50 mg/kg (Kae-M), or 100 mg/kg (Kae-H) Kae (i.p.) for 30 min, prior to LPS challenge (7 mg/kg, i.p.).

### Sample collection

After drug intervention and modeling, approximately 6–8 mL of blood was collected, allowed to clot for 2 h, and centrifuged at 3000 rpm for 20 min. Serum was then transferred to Eppendorf tubes and stored at − 80 °C. Subsequently, rats were euthanized, and heart tissue samples were collected and fixed in 4% paraformaldehyde for 24 h for subsequent histological analysis.

### Hematoxylin and Eosin (HE) analysis

Heart tissues were fixed in 4% paraformaldehyde for 24 h, dehydrated through a graded ethanol series (75%, 85%, 95%, 100%), cleared in xylene, and embedded in paraffin. Section  (5 μm) were deparaffinized, rehydrated through descending graded ethanol, stained with hematoxylin and eosin (Purchased from Zhonghui Hecai Biopharmaceutical Technology Co., Ltd.) for 3 min, differentiated in 1% hydrochloric acid–ethanol for 30 s, washed in running tap water for 15 min, and mounted with neutral balsam. Histopathological changes were examined under an optical microscope (Nikon, Japan) [19].

### H9c2 cell culture

The rat cardiomyocyte cell (H9c2) line was obtained from Wuhan Pricella Biotechnology Co., Ltd. (China). H9c2 cells were cultured in DMEM supplemented with 15% fetal bovine serum and 1% penicillin, and the culture medium was changed every two days. When the cell density reached 90%, trypsin was added, and the cells were collected into centrifuge tubes. After centrifugation, the supernatant was discarded, and the cells were resuspended in fresh medium. The culture was then continued by passaging at a 1: 2 ratio.

### Cell counting Kit-8 (CCK-8) experiment

The rat cardiomyoblast cell line H9c2 was obtained from Wuhan Pricella Biotechnology Co., Ltd. (Wuhan, China). H9c2 cells were cultured in DMEM supplemented with 15% fetal bovine serum and 1% penicillin-streptomycin at 37 °C in a humidified atmosphere with 5% CO₂. The medium was changed every 2 days. Upon reaching ~ 90% confluence, cells were detached with 0.25% trypsin-EDTA, centrifuged at 1000 rpm for 5 min, resuspended in fresh medium, and subcultured at a 1:2 ratio [20].

### Enzyme-linked immunosorbent assay (ELISA)

Cell culture supernatants and serum samples (from in vivo experiments) were collected and centrifuged to remove debris. Concentrations of TNF-α, IL-6, and IL-1β were measured by ELISA according to the manufacturer’s instructions (BOSTER, USA: Rat TNF-α, Cat# EK0526; Rat IL-6, Cat# EK0412; Rat IL-1β, Cat# EK0393), with minor modifications as necessary [21].

### Measurement of SOD, NO, MDA, and GSH-Px expression

The activities of superoxide dismutase (SOD; Cat# A001-3) and glutathione peroxidase (GSH-Px; Cat# A005-1), as well as the levels of nitric oxide (NO; Cat# A013-2-1) and malondialdehyde (MDA; Cat# A003-1), were measured in treated H9c2 cells using commercial kits (Nanjing Jiancheng Bioengineering Institute, Nanjing, China) according to the manufacturer’s instructions [22].

### Reverse transcription-quantitative PCR (RT-qPCR) experiment

Total RNA was extracted from H9c2 cells according to the manufacturer’s instructions, followed by reverse transcription to synthesize complementary DNA. For in vivo experiments, heart tissue was minced and homogenized in 1 mL of Trizol reagent; RNA was then extracted following the same procedure. Subsequently, a PCR reaction mixture was prepared and a thermal cycling protocol was performed to quantify target gene mRNA expression using a fluorescence quantitative PCR instrument [23].

### Protein immunoblot analysis

Cells were seeded at 5 × 10⁴ cells/well in 6-well plates and treated as described. Protein was extracted with RIPA buffer containing protease/phosphatase inhibitors, and the lysate was centrifuged at 12,000×g for 15 min at 4 °C. Protein concentrations were determined by the bicinchoninic acid (BCA) assay. Protein loading amounts varied among samples (10–20 µg per lane). Samples were separated by sodium dodecyl sulfate-polyacrylamide gel electrophoresis (SDS-PAGE) and transferred onto PVDF membranes. After blocking with 5% bovine serum albumin (BSA) for 1 h at room temperature, membranes were incubated with primary antibodies overnight at 4 °C, followed by incubation with an HRP-conjugated secondary antibody for 1 h at room temperature. Signals were detected by enhanced chemiluminescence and quantified with ImageJ 1.54 g. Mean background values were subtracted lane by lane, and target-band intensities were normalized to β-actin. All quantifications were performed independently by two researchers blinded to group allocation [24].

### Immunofluorescence experiment

(1) Cell slides were pre-cleaned and washed prior to use. H9c2 cells were seeded into 24-well plates and, after the designated culture period, culture medium was discarded. Subsequently, 500 µL of 4% paraformaldehyde was added to each well for 15 min. (2) The fixative was removed, and wells were washed with phosphate-buffered saline with Tween-20 (PBST) three times, followed by addition of 500 µL of 0.1% Triton X-100 solution to each well for 15 min at room temperature. (3) The Triton X-100 solution was removed, and wells were washed with PBST three times. Blocking was performed with 500 µL of goat serum for 60 min. Slides were then removed, and primary antibody was added dropwise for overnight incubation at 4 °C. (4) The diluted secondary antibody was added dropwise for 60 min at room temperature in the dark. (5) The secondary antibody was discarded, and wells were washed three times with PBST. DAPI stain was added dropwise for 3 min at room temperature, then discarded, followed by three washes with PBST. (6) Slides were mounted with an antifade mounting medium and stored at 4 °C in the dark. (7) Image acquisition was performed on a Leica TCS SP8 confocal microscope (Leica Microsystems, Germany) using an HC Plan-Apochromat 10×/0.45 NA objective. Single-plane scanning parameters: DAPI (405 nm, 30% laser power, 1250 V gain), Cy3 (488 nm, 15% laser power, 800 V gain); pinhole 1 AU, uniform exposure time 2.43 µs. (8) Image analysis: Images were imported into ImageJ 1.54 g, converted to 8-bit grayscale, and thresholded. Mean gray value was selected in “Set Measurements”, and fluorescence intensity was quantified via the “Measure” command [24].

### Blinding

Regarding the blinding measures in this study, network pharmacology analysis used standard algorithms with preset parameters; data extraction and target prediction were fully automated and objective. For cell experiments, culture plates were randomly grouped at inoculation, and CCK-8 absorbance was collected using a microplate reader. Immunofluorescence images were encoded and analyzed in a blinded manner relative to treatment conditions. During in vivo experiments, researchers performing animal handling, drug administration, and outcome assessments were blinded to group assignments. Kae solutions were prepared and coded by an independent researcher. Tissue samples used for histological and biochemical analyses were labeled with code numbers only.

### Statistical analysis

Data analysis and graphing were conducted using GraphPad Prism version 9.5.0. We employed t-tests and one-way analysis of variance (ANOVA) to evaluate differences between groups. Additionally, a significance level was set at *P* < 0.05 or *P* < 0.01 to determine the statistical significance of the observed group differences (Prior to analysis, data normality was assessed using the Normality and Lognormality Tests. Multiple-group comparisons were performed using one-way ANOVA followed by Tukey’s post-hoc tests. When the normality test failed, nonparametric tests were used). All experiments were performed in triplicate (biological replicates) to ensure reliability.

## Results

### Screening for targets at the intersection of drugs and diseases

**Fig. 1:**
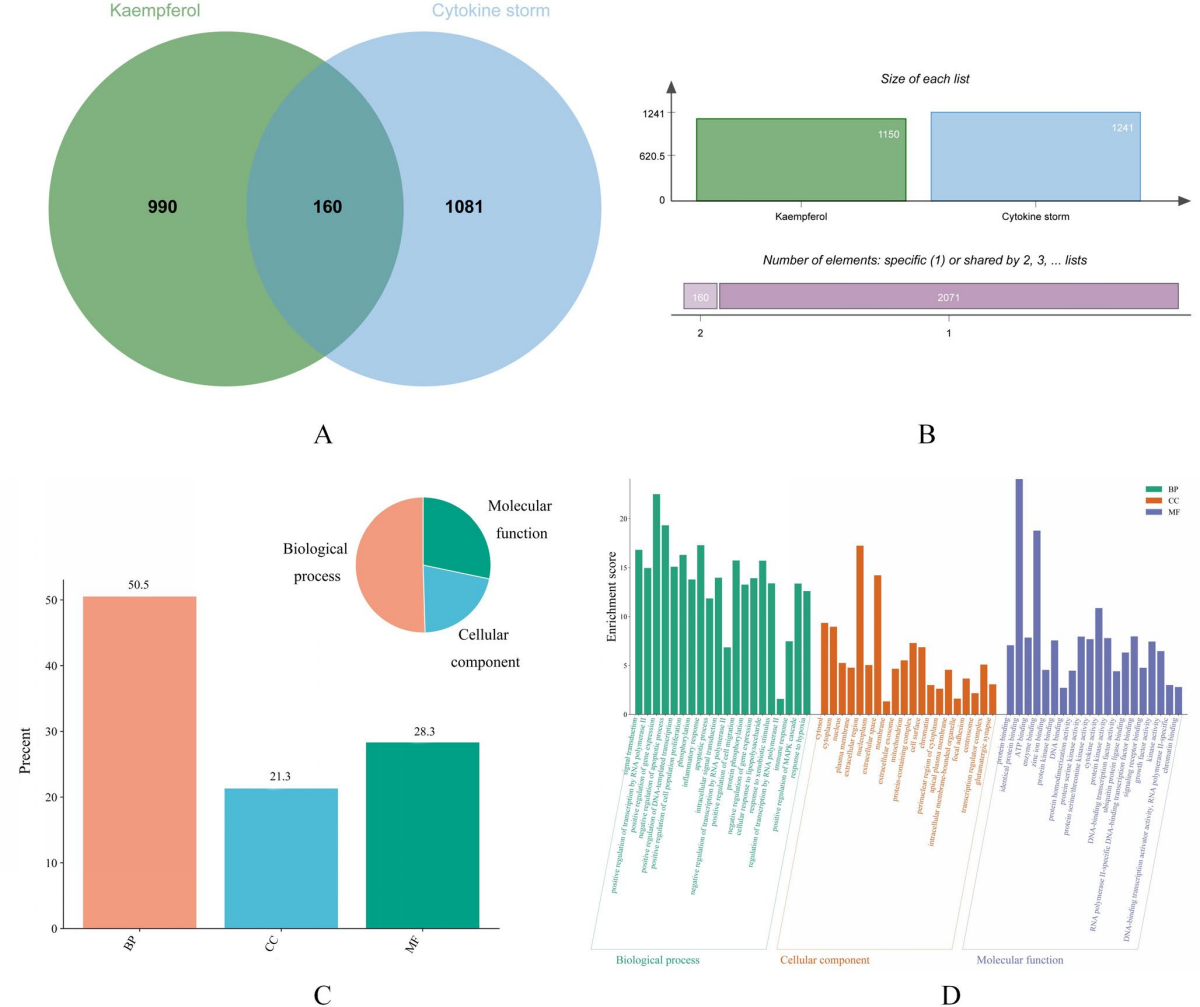
Intersecting targets of kaempferol against cytokine storms Venn diagrams. (**A**) Pie chart of kaempferol (Kae) and cytokine storm (CS) target genes; (**B**) Bar graph illustrating overlapping targets. Gene ontology (GO) Functional Annotation Map. (**C**) Proportions of biological processes (BP), cell component (CC), and molecular function (MF) enrichment analyses; (**D**) top 20 bars of GO enrichment analysis for 160 intersecting targets

This study identified 1,451 Kae-related targets from various databases; after duplicate removal, 1,150 unique targets were retained. A total of 1,297 disease targets were screened from GeneCards (1,238) and OMIM (59) databases. After removing duplicates, 1,241 unique targets were obtained (Fig. 1A). The identified targets of Kae and CS were imported into the Weishengxin platform to identify their intersection, yielding 160 effective targets of Kae for CS. The Kae–CS intersection targets were illustrated in Fig. 1B. Additionally, we used the SWISS ADME database (http://www.swissadme.ch/) to predict Kae’s ADMET (Absorption, Distribution, Metabolism, Excretion, Toxicity) properties. Results showed that Kae exhibited high gastrointestinal absorption and a drug-likeness score ≥ 2, with favorable pharmacokinetic properties and bioavailability.

### GO analysis of drug-disease intersection targets

GO analysis results are shown in Fig. 1C and D. Among them, the terms significantly enriched in biological processes (BP) included signal transduction, phosphorylation, inflammatory response, and other processes. The terms significantly enriched in cellular components (CC) included the extracellular region, extracellular space, cytoplasm, nucleus, and other cellular components. The terms significantly enriched in molecular function (MF) included identical protein binding, enzyme binding, cytokine activity, protein kinase binding, and other molecular functions.

### KEGG pathway enrichment analysis of drug-disease intersection targets

**Fig. 2:**
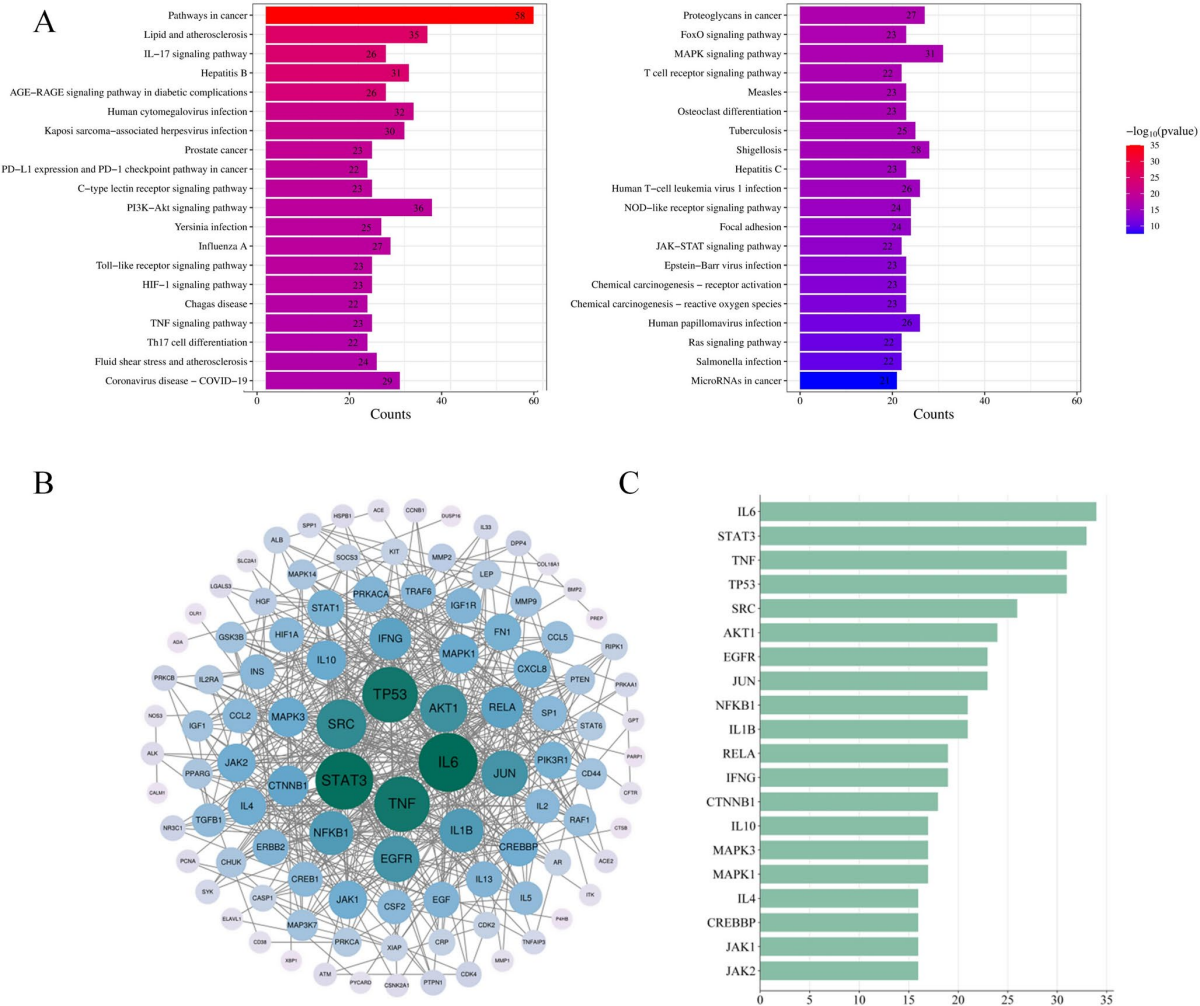
(**A**) A map of the kyoto encyclopedia of genes and genomes (KEGG) signaling pathway in the anti-cytokine storm intersection target of kaempferol. Numbers on the bars represent COUNTS values. Legend source: www.kegg.jp/kegg/kegg1.html. PPI network diagram of the interaction between kaempferol and cytokine storm intersection targets. (**B**) Protein-protein interaction (PPI) network diagram; (**C**) Core targets bar chart. The darker the color, the larger the “degree”value

The KEGG pathway enrichment analysis indicated that the mechanism by which Kae contributes to the prevention and treatment of CS may involve 187 unique signaling pathways. Using a predefined criterion, we screened 40 signaling pathways to explore the mechanisms underlying the protective effects of Kae against CS. The results are presented in Fig. 2A. After excluding pathways associated with cancer and other diseases, we found that the specific mechanism of Kae in preventing and treating CS may be closely related to the PI3K-AKT, JAK-STAT, IL-17, and AGE-RAGE signaling pathways, among others.

### PPI network construction for intersecting targets and screening of core targets

The 160 intersecting targets were imported into the STRING database, and the protein interaction network between Kae and CS was mapped accordingly (see Fig. 2B for details). In this network diagram, the node centrality, edge number, node size, and color shade are all proportional to the likelihood of a protein being a core target. Subsequently, these targets were analyzed in depth using Cytoscape 3.9.0 software, and bar charts were constructed for the top 20 core targets ranked by the number of adjacent nodes in descending order (Fig. 2C).

### GO and KEGG pathway enrichment analysis of core targets

The 20 core targets identified were re-imported into the DAVID database for KEGG pathway enrichment analysis and GO functional annotation. The results are presented in **Supplementary Fig. 1A–1D**. BP analysis primarily revealed enrichment in cellular responses to lipopolysaccharides, protein phosphorylation, and inflammatory responses. CC analysis indicated that Kae’s targets were predominantly localized in the cytosol, cytoplasm, and nucleus. In addition, KEGG pathway enrichment analysis indicates that the protective effect of Kae on CS potentially involves the JAK-STAT and PI3K-AKT signaling pathways.

### Molecular docking results

Based on the degree value distribution of core targets in the PPI network and several highly significant pathways identified in the KEGG enrichment analysis, IL-6, JAK2, and STAT3 were ultimately selected for in-depth investigation. It is generally accepted that lower binding energy indicates stronger affinity between the receptor and ligand, thereby increasing the likelihood of binding. Through molecular docking, we found that Kae exhibited significant interactions with these pathway proteins. Docking scores are reported in kcal/mol; generally, binding energies ≤ − 5.0 kcal/mol indicate moderate binding affinity, whereas energies ≤ − 7.0 kcal/mol indicate strong binding affinity [25–27].

In our study, the binding energies of Kae to IL-6, JAK2, and STAT3 were − 7.4, − 9.3, and − 7.9 kcal/mol, respectively. Within the IL-6 receptor, Kae formed hydrogen bonds with ARG-169, LEU-63, and LEU-65. In the JAK2 receptor, hydrogen bonds were formed with LEU-932, GLY-993, and LYS-857. In the STAT3 receptor, hydrogen bonds were observed with THR-440, ASP-369, LEU-438, and ARG-379. These results indicate that Kae exhibits strong binding affinity for IL-6, JAK2, and STAT3. The molecular docking visualizations are presented in Supplementary Fig. 2A–2C, demonstrating that the binding between Kae and these three receptor proteins is both stable and specific.

### The effects of kae on cardiac tissue in rats with cytokine storm

**Fig. 3:**
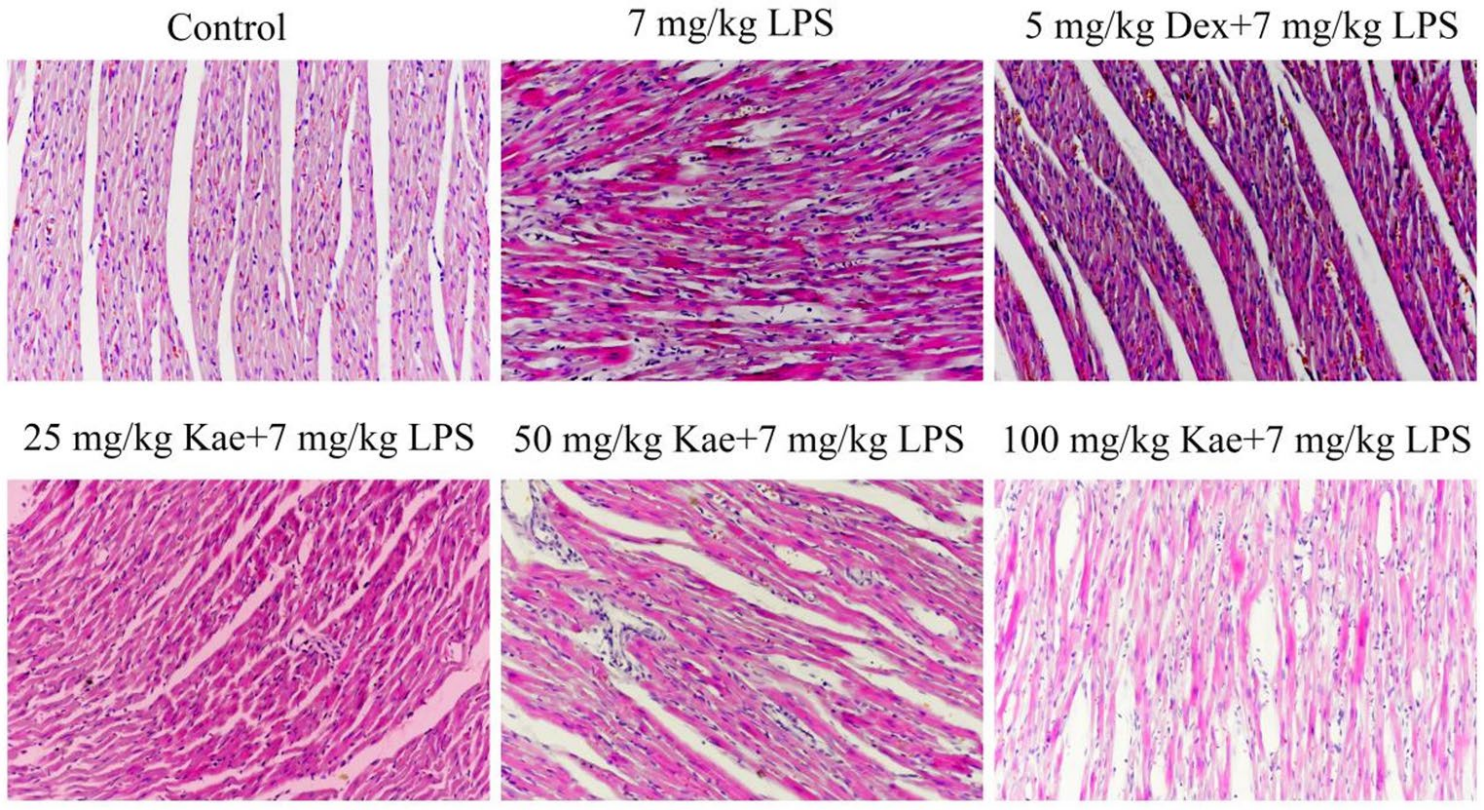
Representative H&E staining images of myocardial tissue sections (*n* = 6 per group; magnification, ×200)

HE staining of rat heart tissue showed that myocardial fibers in the control group were orderly arranged with abundant sarcoplasm. In contrast, the model group exhibited fragmented myocardial fibers and obvious congestion in the connective tissue of the myocardial interstitium, accompanied by inflammatory cell infiltration leading to myocardial necrosis. However, Kae pretreatment significantly ameliorated these pathological changes. This result strongly indicates that Kae exerts a significant protective effect against the inflammatory response in rat myocardium and effectively attenuates inflammatory damage to myocardial tissue (Fig. 3).

### The effects of kae on the expression of pro-inflammatory factors in rat serum and cardiac muscle tissue

To further determine whether Kae pretreatment can suppress inflammatory responses, serum levels of proinflammatory factors were measured across groups. As shown in **Supplementary Fig. 3A–3C**, serum inflammatory factor levels in the model group were significantly higher than those in the control group (*P* < 0.01), indicating that LPS could effectively induce systemic inflammatory responses in rats. Compared with the model group, both Kae- and Dex-pretreated groups showed reduced serum levels of IL-6, IL-1β, and TNF-α (*P* < 0.01).

In addition, compared with the Con group, mRNA expression levels of IL-6, IL-1β, and TNF-α were significantly increased after LPS stimulation (*P* < 0.01). In contrast, compared with the model group, mRNA expression levels of these three proinflammatory factors were significantly decreased following pretreatment with Dex or Kae at various concentrations (*P* < 0.01). These results are shown in Supplementary Fig. 3D–3F.

### Screening of safe action concentrations of kae and optimal modeling concentrations for an inflammatory response model

**Fig. 4:**
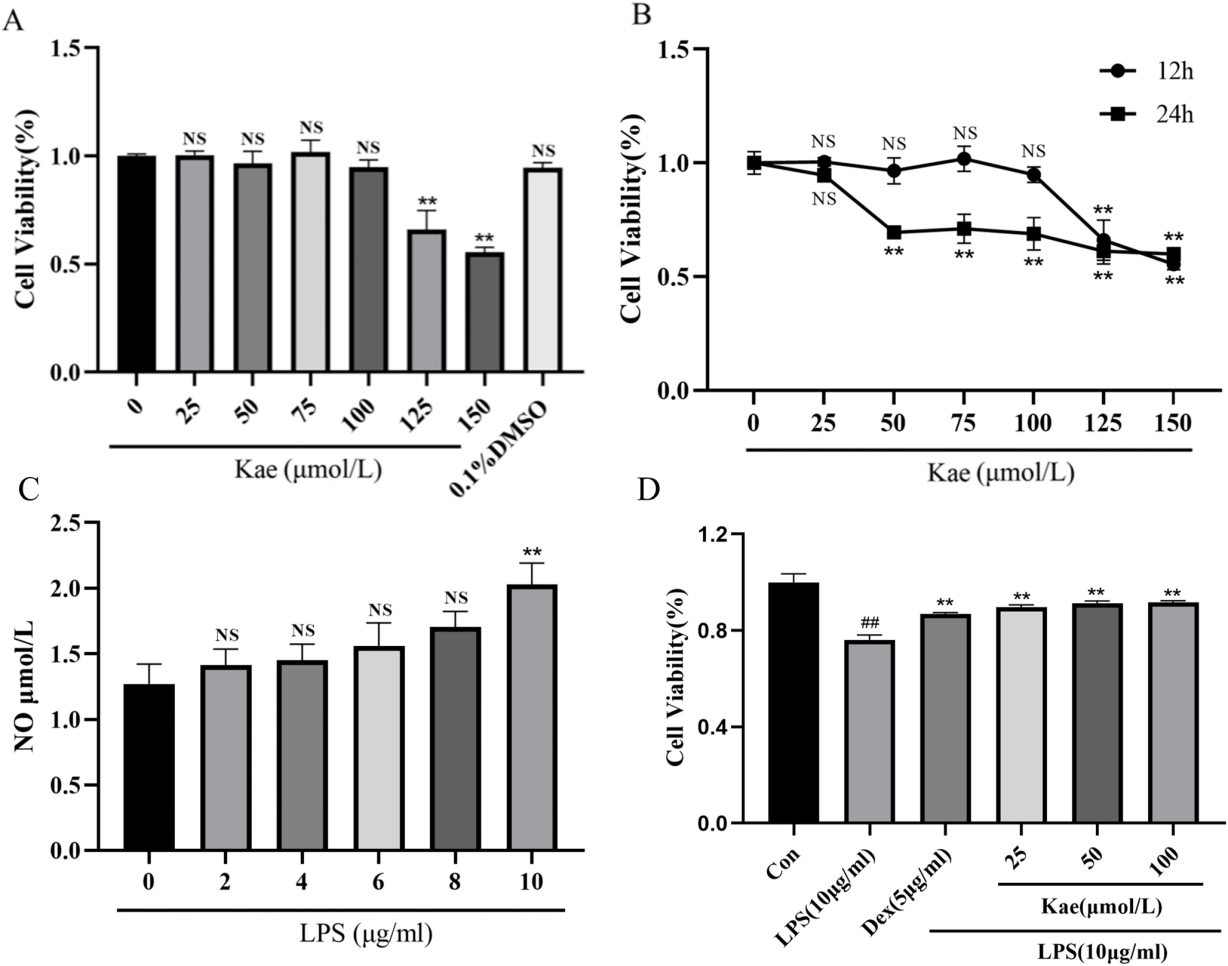
Effect of Kae on H9c2 cell viability at different concentrations and at different time points. (**A**) 12 h; (**B**) 12 h VS 24 h. (**C**) Effect of different concentrations of lipopolysaccharide (LPS) on nitric oxide (NO) content after 12 h of intervention in H9c2 cells. (**D**) Effect of Kae and LPS on H9c2 cell viability after 12 h of co-action. NS: Not Statistically, ^**^P < 0.01 vs. 0 µmol/L group or Control group. Data represent *n* = 3 biological replicates per group

To determine the safe and effective concentration of Kae, we investigated its effects on H9c2 cell viability at various concentrations over 12 and 24 h using the CCK-8 assay. As shown in Fig. 4A, H9c2 cells were treated with 25, 50, 75, 100, 125, and 150 µmol/L Kae for 12 h to assess cell viability. The results indicated that Kae at concentrations ≤ 100 µmol/L had no significant effect on H9c2 cell viability (*P* > 0.05). However, H9c2 cell viability was significantly reduced at concentrations > 100 µmol/L (*P* < 0.01). Moreover, it was evident from the figure that 0.1% DMSO did not affect H9c2 cell viability. To further assess optimal concentrations of Kae for intervention, we treated H9c2 cells with the same concentration range for an additional 24 h. As shown in Fig. 4B, H9c2 cell viability significantly decreased after 24 h compared with 12 h at the same concentrations. Therefore, H9c2 cells were treated with Kae at 25 µmol/L (low dose), 50 µmol/L (medium dose), and 100 µmol/L (high dose) for 12 h in subsequent experiments.

NO levels in cell culture supernatant indicate the severity of cellular inflammation. Therefore, NO measurement can help verify whether the inflammatory response model of H9c2 cells was successfully established. Based on relevant literature [28, 29], H9c2 cells were treated with LPS at concentrations of 0 (control), 2, 4, 6, 8, and 10 µg/mL for 12 h, after which NO levels were measured (Fig. 4C). The results indicated that, compared with the control group (0 µg/mL), NO levels were significantly elevated at 10 µg/mL LPS (*P* < 0.01). Therefore, optimal conditions for the LPS-induced inflammatory response in H9c2 cells were determined to be 10 µg/mL LPS for 12 h.

### Effect of the combined action of kae and LPS on the viability of H9c2 cells

After inducing inflammatory responses in H9c2 cells, we further investigated the effects of Kae pretreatment at various concentrations in combination with LPS on cell viability after 12 h. The results, illustrated in Fig. 4D, indicated that LPS significantly reduced H9c2 cell viability (*P* < 0.01). However, pretreatment with Dex or Kae at various concentrations significantly enhanced cell viability (*P* < 0.01).

### Effect of kae on LPS-induced expression of inflammatory factors in H9c2 Cells

The results are presented in Supplementary Fig. 4A–4C. Levels of IL-6, IL-1β, and TNF-α in H9c2 cell culture supernatant were significantly elevated compared with the control group following LPS stimulation (*P* < 0.01). Secretion levels of these cytokines were significantly reduced following pretreatment with Kae or Dex. It was evident that the high-dose Kae pretreatment group exhibited the strongest inhibitory effect compared with the medium- and low-dose groups. Although the specific mechanism remained unclear, Kae pretreatment significantly reduced LPS-induced secretion of IL-6, IL-1β, and TNF-α, as measured by ELISA, suggesting attenuation of the inflammatory response in H9c2 cells.

### Effect of kae on LPS-induced oxidative stress markers in H9c2 cells

Inflammation and oxidative stress are closely interrelated. Therefore, we examined the effects of Kae pretreatment on oxidative stress indicators, including MDA, SOD, GSH-Px, and NO. The results are presented in Supplementary Fig. 4D–4G. Following LPS treatment, MDA and NO levels increased significantly, whereas SOD activity decreased significantly. Kae pretreatment effectively inhibited the elevation of these markers and enhanced SOD activity, suggesting that Kae could ameliorate LPS-induced inflammatory response and oxidative stress in H9c2 cells.

### Effect of kae on mRNA expression levels of IL-6, IL-10, JAK2, and other cytokines

**Fig. 5:**
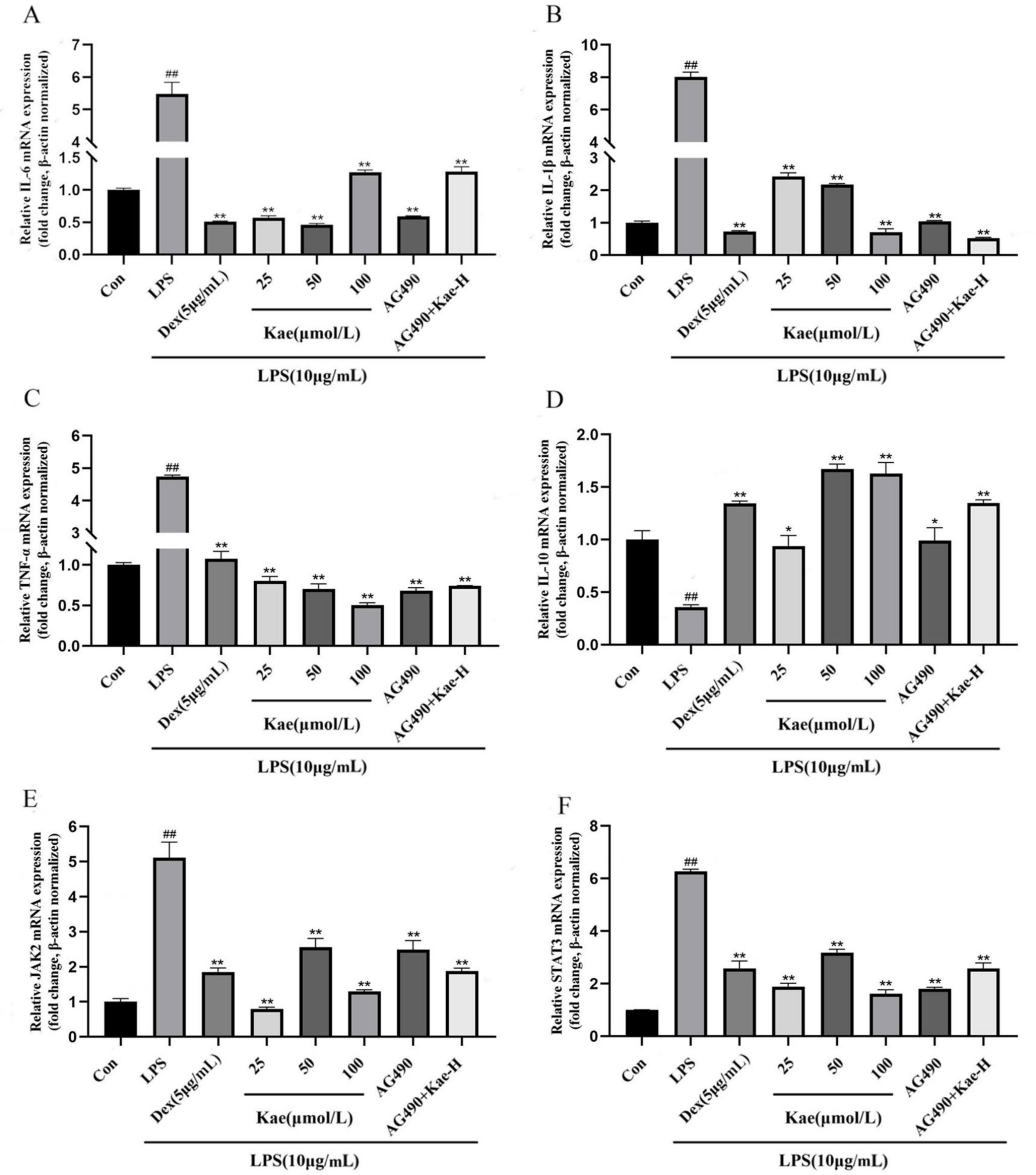
Effect of Kae on the mRNA expression of IL-6, IL-1β, TNF-α, IL-10, JAK2, and STAT3. (**A**) Interleukin-6 (IL-6); (**B**) Interleukin-1β (IL-1β); (**C**) Tumor necrosis factor alpha (TNF-α); (**D**) Interleukin-10 (IL-10); (**E**) Ianus kinase 2 (JAK2); (**F**) Signal transducer and activator of transcription 3 (STAT3). ^##^*P* < 0.01 vs. Control group, ^*^P < 0.05, ^**^P < 0.01 vs. LPS group. Data represent *n* = 3 biological replicates per group

To assess the anti-inflammatory effects of Kae, we evaluated the impact of Kae pretreatment on mRNA expression levels of proinflammatory cytokines, including IL-6, IL-1β, and TNF-α, using RT-qPCR. The results are presented in Fig. 5. LPS treatment significantly increased mRNA levels of proinflammatory cytokines (IL-6, IL-1β, and TNF-α) and signaling molecules (JAK2 and STAT3), while reducing expression of the anti-inflammatory factor IL-10. Both Kae and AG490 pretreatment decreased mRNA expression of these cytokines while increasing IL-10 expression. This finding was fully consistent with ELISA results. However, further analysis indicated that AG490 alone reduced cytokine mRNA expression, whereas this effect was attenuated by co-treatment with Kae-H.

### Effect of kae on the expression of JAK2, STAT3, p-JAK2, and p-STAT3 in LPS-induced H9c2 cells

**Fig. 6:**
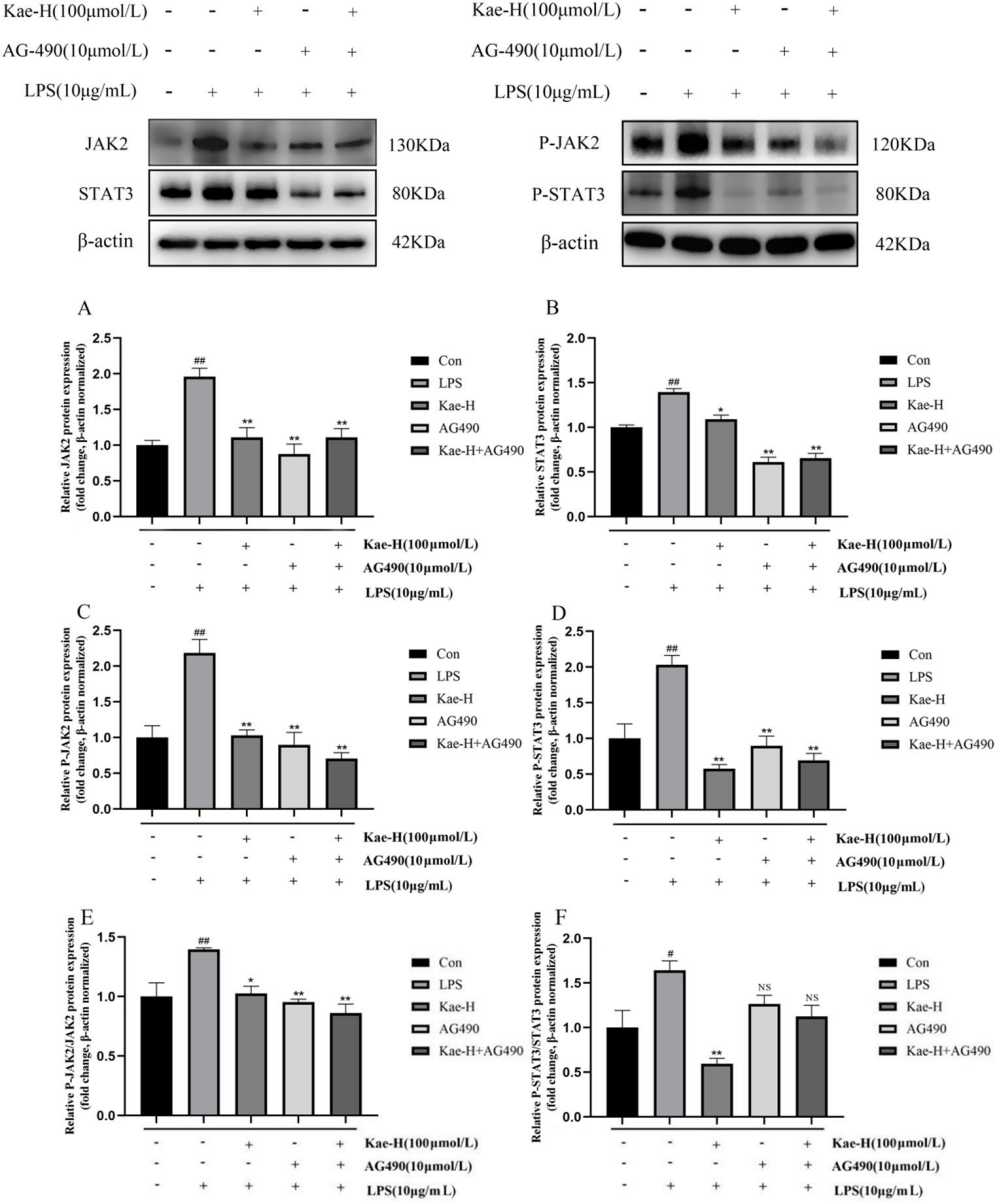
Effect of Kae on the expression of JAK2, STAT3, p-JAK2, and p-STAT3 proteins. (**A**) JAK2; (**B**) STAT3; (**C**) p-JAK2; (**D**) p-STAT3; (**E**) p-JAK2/JAK2; (**F**) p-STAT3/STAT3. NS: Not Statistically, ^#^*P* < 0.05, ^##^*P* < 0.01 vs. Control group, ^*^P < 0.05, ^**^P < 0.01 vs. LPS group. Data represent *n* = 3 biological replicates per group

To investigate the mechanism by which Kae pretreatment alleviated LPS-induced inflammation in H9c2 cells, we analyzed the effects of Kae on the IL-6/JAK2/STAT3 signaling pathway using western blotting. Based on the aforementioned results, the Kae-H pretreatment group demonstrated significant therapeutic efficacy. Therefore, this section focused solely on the high-dose Kae pretreatment group to evaluate the impact of Kae on expression of JAK2, STAT3, p-JAK2, and p-STAT3 proteins. The results are presented in Fig. 6. Compared with the control group, LPS treatment significantly increased expression of JAK2, STAT3, p-JAK2, and p-STAT3 proteins (*P* < 0.01). Following pretreatment with Kae-H or AG490, expression of all four proteins was significantly reduced (*P* < 0.05). Overall, the inhibitory effects of Kae-H and AG490 were comparable. Western blotting results were consistent with those of RT-qPCR, indicating that Kae pretreatment alleviated LPS-induced inflammatory response in H9c2 cells. This effect potentially involved modulation of the IL-6/JAK2/STAT3 signaling pathway.

To accurately reflect changes in phosphorylation levels and provide comprehensive biological information, we examined the effects of Kae on the expression of p-JAK2/JAK2 and p-STAT3/STAT3 proteins. Compared to the control group, the levels of p-JAK2/JAK2 and p-STAT3/STAT3 proteins in the LPS group exhibited a significant increase (*P* < 0.05). Following pretreatment with Kae-H and AG490, the expression levels decreased markedly (*P* < 0.05). Notably, the ratio of p-JAK2/JAK2 exhibited a more pronounced reduction.

**Fig. 7:**
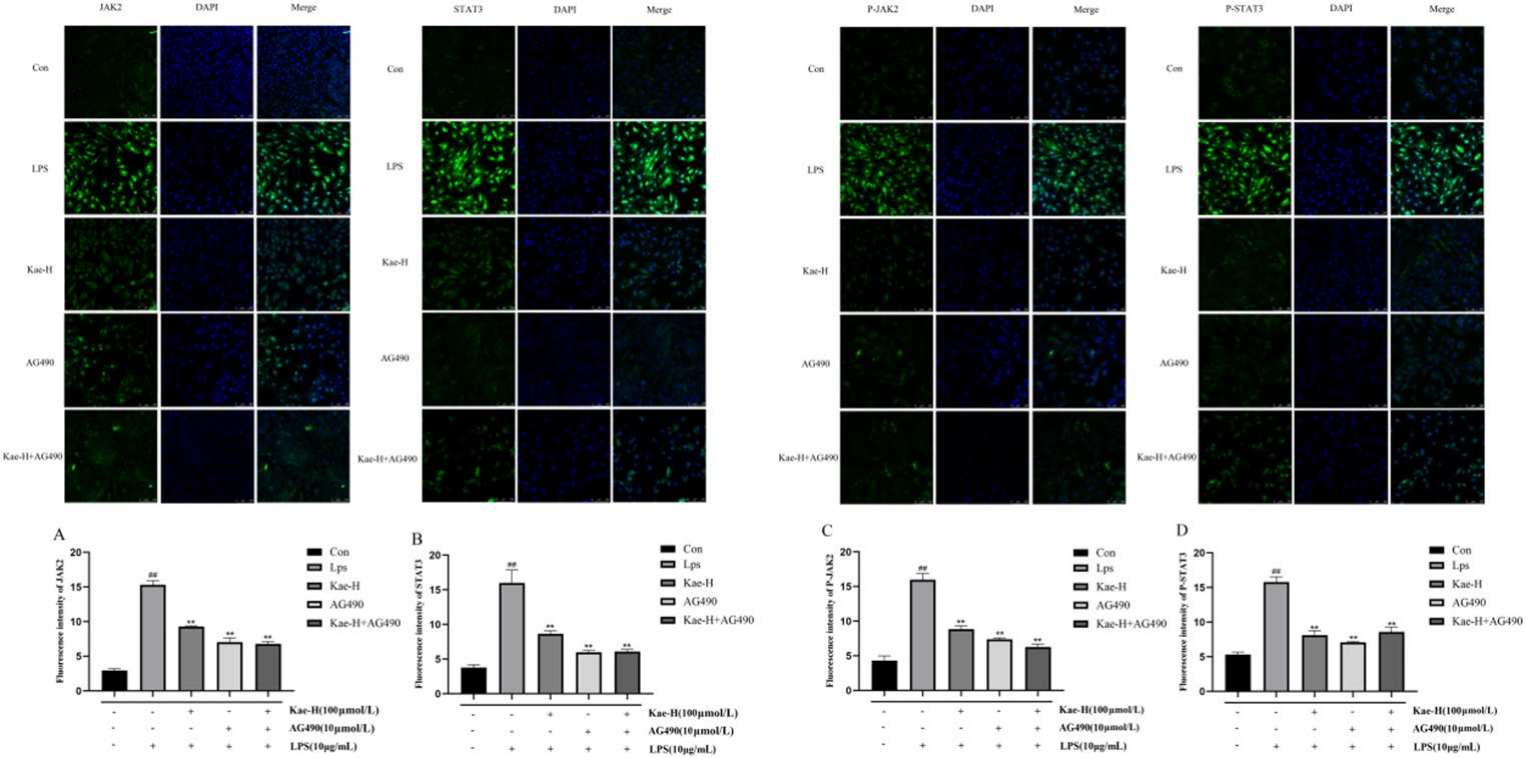
The effects of Kae on the expression of JAK2, STAT3, p-JAK2, and p-STAT3 protein levels were explored by immunofluorescence experiments. (**A**) JAK2; (**B**) STAT3; (**C**) p-JAK2; (**D**) p-STAT3. DAPI: blue. Mean fluorescence intensity per cell was quantified using ImageJ, normalized to control (set as 1.0), and expressed as fold change. Scale bar: 100 μm. ^##^*P* < 0.01 vs. Control group, ^**^P < 0.01 vs. LPS group. Data represent *n* = 3 biological replicates per group

In addition, to visually demonstrate the effects of Kae on protein expression within the IL-6/JAK2/STAT3 signaling pathway, we employed immunofluorescence staining to examine JAK2, STAT3, p-JAK2, and p-STAT3 expression. As illustrated in Fig. 7, fluorescence intensities of JAK2, STAT3, p-JAK2, and p-STAT3 in the LPS group were significantly higher than those in the control group (*P* < 0.01). This finding was consistent with Western blotting results, indicating that LPS could activate the JAK2/STAT3 signaling pathway in H9c2 cells. Fluorescence intensities of JAK2, STAT3, p-JAK2, and p-STAT3 in Kae-H and AG490 pretreatment groups were significantly lower than those in the LPS group (*P* < 0.01). This indicated that Kae and AG490 had comparable inhibitory effects on expression of JAK2, STAT3, p-JAK2, and p-STAT3.

## Discussion

CS is a complex pathological process triggered by multiple factors. Within the CS milieu, massive production of IL-6 and IL-1β excessively activates immune cells and amplifies the cytokine cascade, thereby exerting destructive effects on the myocardium [30]. Therefore, inhibiting excessive release of proinflammatory cytokines, such as IL-6 and IL-1β, is crucial for controlling CS. Our findings indicated that Kae effectively inhibited expression and secretion of IL-6, IL-1β, and TNF-α in rat serum and cardiac tissue, as well as in H9c2 cardiomyocytes, thereby preventing CS onset. Notably, the anti-CS activity of Kae exhibited no clear dose dependency. Nevertheless, considering all indicators, high-dose Kae demonstrated relatively potent anti-CS effects.

Kae showed efficacy in multiple inflammation-driven diseases. Previous studies demonstrated that Kae modulated multiple cellular signaling pathways to suppress chemokines and proinflammatory mediators (Supplementary Table 1) [31–35]. In summary, the inhibitory effects of Kae on proinflammatory factors (such as IL-6 and IL-1β) are highly consistent with the regulatory mechanisms of CS. Interestingly, however, we observed higher IL-6 and STAT3 mRNA expression levels in the Kae-H and AG490 co-treatment group compared with the AG490 monotherapy group. Reviewing relevant literature, we found that suppressor of cytokine signaling 3 (SOCS3), a classic negative feedback regulator of JAK2, may diminish the inhibitory effect of AG490 on JAK2 [36]. This may be related to the timing of the action of Kae, as hydroxyflavone A, a structurally related flavonoid, has been shown to induce SOCS3-mediated negative feedback signaling within 3 h, thereby reducing the efficacy of JAK2 inhibitors, whereas it shows no effect on SOCS3 expression at 6 and 9 h [37]. Nevertheless, our study quantified transcriptional expression of proinflammatory cytokines (IL-6) and signaling molecules (STAT3), providing molecular evidence for CS pathogenesis and the therapeutic efficacy of Kae.

Following oral administration, Kae undergoes extensive first-pass metabolism, forming glucuronide and sulfate conjugates in plasma. These conjugates exhibit reduced biological activity compared with free Kae, thereby contributing to its poor oral bioavailability [38]. However, due to its lipophilic nature, Kae demonstrates good cellular uptake in cardiomyocytes, undergoes passive diffusion across cell membranes, and is slowly metabolized in H9c2 cells [39, 40]. Previous studies have reported that Kae significantly inhibits production of inflammatory cytokines, such as TNF-α and IL-6, at concentrations of 5–100 µmol/L in cell models, supporting the selection of 25, 50, and 100 µM as experimental concentrations for subsequent experiments [16, 41]. Nevertheless, the translational relevance of these in vitro concentrations requires thorough evaluation against human pharmacokinetic data. For example, caution is required when Kae is co-administered with drugs metabolized by cytochrome P450 2C9 (CYP2C9), because Kae is a potent mechanism-based inhibitor of this enzyme and can significantly alter pharmacokinetic characteristics of CYP2C9 substrates, thus posing potential interaction risks [42]. Despite these pharmacokinetic concerns, Kae demonstrated a relatively good safety profile in humans. A recent randomized, double-blind, placebo-controlled trial demonstrated that Kae aglycone at 50 mg/day (approximately five times the average dietary intake) was well tolerated by healthy adults for four weeks, with no clinically significant adverse reactions observed [43]. However, we acknowledge that evidence directly supporting its efficacy in cardiovascular disease treatment remains limited, primarily due to a lack of large-scale randomized controlled trials.

Bioinformatics analysis results demonstrated that the potential therapeutic effects of Kae on CS primarily target the PI3K/AKT, IL-17, and JAK/STAT signaling pathways. Although our experimental validation focused on the JAK2/STAT3 pathway, it should be noted that other pathways identified by bioinformatics analysis may also contribute to cardioprotective effects of Kae. The PI3K/AKT pathway is a recognized regulator of cardiomyocyte survival and anti-apoptotic signaling, and Kae-mediated activation of this pathway has been confirmed in other cell types [44]. Similarly, IL-17 signaling critically amplifies inflammatory cascades in cardiovascular diseases, and its predicted targeting by Kae warrants further investigation [45]. The selective validation of JAK2/STAT3 in this study was based on the highest enrichment significance in our KEGG analysis and its specific relevance to LPS-induced inflammatory responses in cardiomyocytes. However, we recognized that these pathways are not mutually exclusive, and crosstalk between JAK2/STAT3 and PI3K/AKT signaling has been documented in cardiac inflammation models [46]. Future studies employing multi-pathway inhibition and transcriptomic profiling are necessary to delineate the relative contributions and interactive dynamics of these signaling networks in Kae-mediated cardioprotection.

**Fig. 8:**
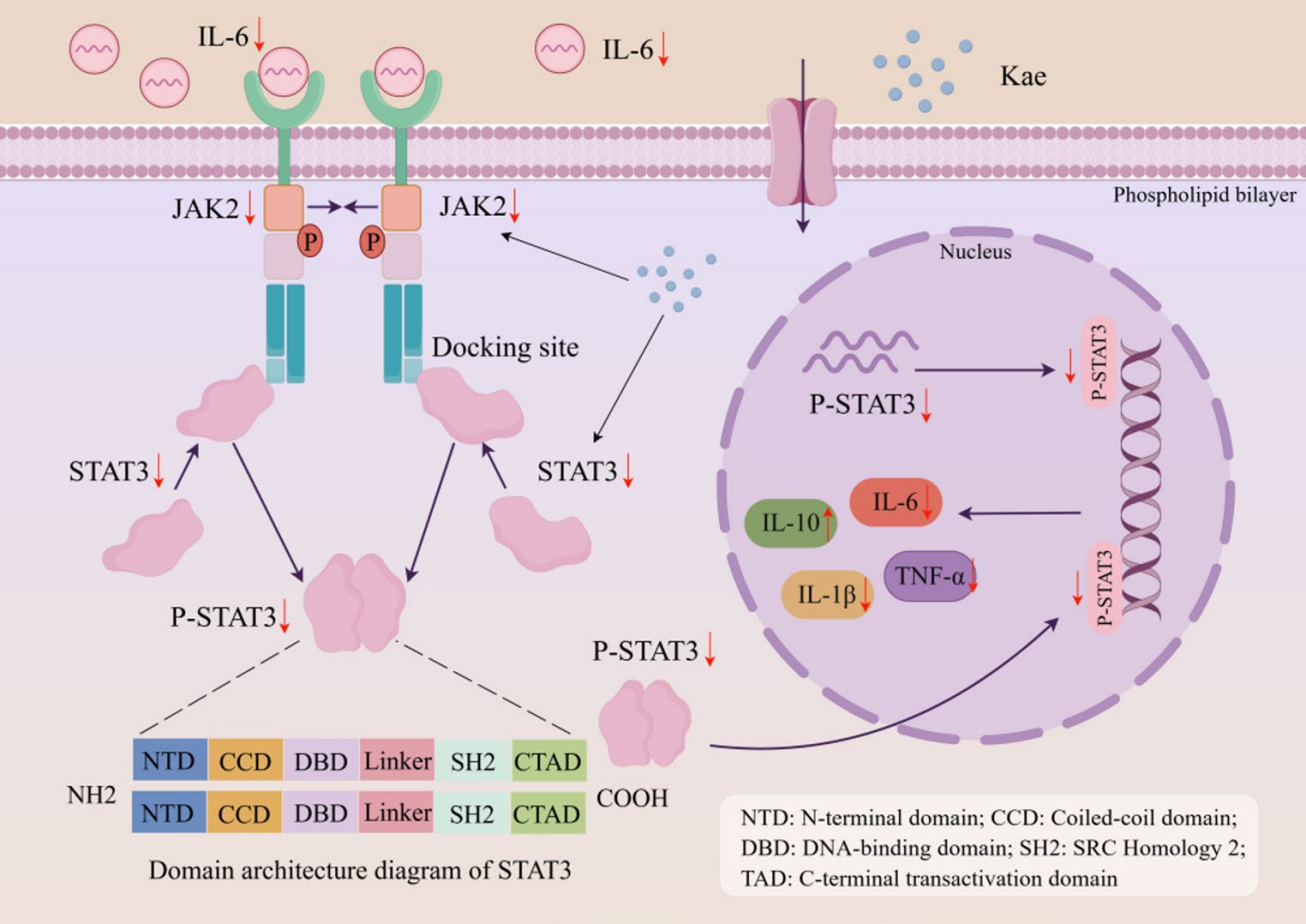
Specific mechanism of action of kaempferol in modulating the IL-6/JAK2/STAT3 signaling pathway to attenuate the inflammatory response. This diagram was created by Figdraw.com

The IL-6/JAK2/STAT3 signaling pathway regulates CS by transducing signals from various cytokines [47]. The activation of this pathway stimulates NF-κB and upregulates adhesion molecule expression, thereby promoting release of inflammatory factors such as TNF-α, IL-6, and IL-1β [48, 49]. Elevated inflammatory cytokine expression can positively feedback to activate NF-κB, ultimately driving inflammatory response onset and compromising normal tissue and cellular physiological functions [50]. In CS, the IL-6/JAK2/STAT3 signaling pathway plays a dual role as both an amplifier and a sustainer. Its overactivation is a key factor underlying the dysregulation of systemic inflammation. Targeting this pathway can disrupt the harmful IL-6–STAT3–IL-6 feedback loop within hours. This strategy is recommended by multiple clinical guidelines for the treatment of CS [51]. By integrating high-confidence PPI targets with molecular docking results, we clarify the role of the IL-6/JAK2/STAT3 pathway in the action of Kae against CS. Our experimental results confirm that Kae is potentially involved in inhibiting JAK2 phosphorylation and downstream STAT3 activation (similar to AG490), thereby protecting cardiomyocytes from CS-induced injury. Furthermore, the Kae concentration used in the present study overlaps with previously reported levels in rat myocardial tissue, underscoring its translational relevance [52]. The specific regulatory effects of Kae’s anti-CS properties are illustrated in Fig. 8.

It is well known that oxidative stress plays an irreplaceable role in the development of many diseases. During heart failure, oxidative stress can lead to an excessive production of NO. When the level of NO exceeds the buffering capacity of the antioxidant defense system, it can cause damage to, and death of, myocardial cells, thereby inducing the progression of heart failure [53]. In addition, large amounts of oxidative-stress products can continuously activate JAK2, leading to massive release of IL-6, TNF-α, and other substances, thereby further increasing the production of oxidative stress products and creating a vicious oxidation-inflammation cycle exacerbating CS [54]. However, we find that Kae alleviates oxidative stress and disrupts the resulting feedback loop by enhancing SOD activity and regulating NO, MDA, and GSH-Px levels.

## Limitations and future perspectives

CS spans multiple disciplines, including infection, oncology, rheumatology, transplantation, and critical care, and myocarditis caused by CS is a fatal commonality among many acute and critically ill patients. CS represents an extreme manifestation of the immune system imbalance, and in-depth in vitro studies of it serve as a bridge connecting basic immunology and clinical applications. Although the anti-inflammatory effects of Kae have been reported in various cellular contexts, it remains unclear whether it can regulate the JAK2/STAT3 axis to alleviate myocarditis caused by CS. This study elucidates the role of Kae in CS-induced myocardial inflammation and its underlying mechanisms, providing not only strategies for the prevention and treatment of CS but also a theoretical foundation for the modern application of traditional medicine.

However, the study has certain limitations. First, the validation of this mechanism is not sufficient, as no JAK2/STAT3 gene knockdown models were employed to evaluate the effects of Kae. Second, research on Kae-mediated inhibition of oxidative stress in H9c2 cells remains at an early stage, and our current analysis is relatively superficial. The interaction between ROS and JAK2 signaling following Kae treatment was not thoroughly investigated herein. To comprehensively elucidate the biological function of Kae, particularly its antioxidant role, future research warrants further depth, specifically detailed experimental validation of Kae’s antioxidant mechanisms and assessment of dose- and time-dependent effects on oxidative stress in H9c2 cells. In addition, the 100 µmol/L kaempferol concentration selected based on 12-h cytotoxicity screening is methodologically sound, yet its physiological relevance is questionable, as achieving such systemic levels in vivo is pharmacologically implausible. Finally, H9c2 cells, an embryonic rat cardiomyocyte line, lack human myocardial metabolic profiles, electrophysiological properties, and immune-cell interactions, limiting their translational fidelity to human pathophysiology; extrapolation of our findings to clinical settings must therefore be undertaken with caution.

Moving forward, we plan to validate our findings through three complementary approaches: (1) genetic confirmation using cardiomyocyte-specific JAK2/STAT3 knockout mouse models to establish the on-target mechanism; (2) pharmacological confirmation using the JAK2 activator coumermycin A1 to validate the anti-inflammatory effects of Kae via the JAK2/STAT3 signaling pathway; (3) clinical translation via prospective cohort studies and/or Phase I/II trials investigating the adjunctive use of Kae in patients with elevated IL-6 levels.

## Conclusion

In summary, this study first utilized network pharmacology and molecular docking techniques to predict the potential mechanisms of action of Kae in CS. Subsequently, in vivo experiments were conducted to investigate the protective effects of Kae pretreatment against CS-induced myocardial inflammation. Finally, in vitro experiments further validated the effects of Kae on JAK2 and STAT3, key proteins of this pathway, thereby demonstrating that the protective effect of Kae against CS potentially involves regulation of the IL-6/JAK2/STAT3 signaling pathway.

## Electronic Supplementary Material

Below is the link to the electronic supplementary material.

Supplementary Material 1

Supplementary Material 2

Supplementary Material 3

Supplementary Material 4

Supplementary Material 5

Supplementary Material 6

## Data Availability

The raw data shown in this study have been provided in the article or supplementary material. Of course, you can also contact the appropriate authors (Hongyi Yue or Wenhua Li) and we will provide the data upon request.

## Abbreviations

Kae: Kaempferol
LPS: Lipopolysaccharide
IL-6: Interleukin-6
IL-1β: Interleukin-1β
TNF-α: Tumor necrosis factor alpha
JAK2: Janus kinase 2
STAT3: Signal transducer and activator of transcription 3
p-JAK2: Phosphorylated Janus kinase 2
p-STAT3: Phosphorylated signal transducer and activator of transcription 3
GO: Gene ontology
KEGG: Kyoto encyclopedia of genes and genomes
SOD: Superoxide dismutase
NO: Nitric oxide
MDA: Malondialdehyde
GSH-Px: Glutathione peroxidase
CCK-8: Cell counting kit-8
ELISA: Enzyme-linked immunosorbent assay
RT-qPCR: Reverse transcription-quantitative PCR
PVDF: Polyvinylidene fluoride
PBST: Phosphate buffered saline with tween
DAPI: 4’,6-diamidino-2-phenylindole
SDS-PAGE: Sodium dodecyl sulfate-polyacrylamide gel electrophoresis

## References

[1] Kim JS, Lee JY, Yang JW, Lee KH, Effenberger M, Szpirt W, Kronbichler A, Shin JI (2021) Immunopathogenesis and treatment of cytokine storm in COVID-19. Theranostics 11(1):316–329 33391477 10.7150/thno.49713 PMC7681075

[2] Chang C, Hu L, Sun S, Song Y, Liu S, Wang J, Li P (2021) Regulatory role of the TLR4/JNK signaling pathway in sepsis–induced myocardial dysfunction. Mol Med Rep 23(5):334 10.3892/mmr.2021.11973 10.3892/mmr.2021.11973 PMC7974310 33760172

[3] Burki TK (2018) Sharp rise in sepsis deaths in the UK. Lancet Respir Med 6(11):826 30219243 10.1016/S2213-2600(18)30382-5

[4] Singer BH, Dickson RP, Denstaedt SJ, Newstead MW, Kim K, Falkowski NR, Erb-Downward JR, Schmidt TM, Huffnagle GB, Standiford TJ (2018) Bacterial Dissemination to the Brain in Sepsis. Am J Respir Crit Care Med 197(6):747–756 29232157 10.1164/rccm.201708-1559OC PMC5855074

[5] Ginestra JC, Coz Yataco AO, Dugar SP, Dettmer MR (2024) Hospital-Onset Sepsis Warrants Expanded Investigation and Consideration as a Unique Clinical Entity. Chest 165(6):1421–1430 38246522 10.1016/j.chest.2024.01.028 PMC11177099

[6] Ren J, Lu Y, Qian Y, Chen B, Wu T, Ji G (2019) Recent progress regarding kaempferol for the treatment of various diseases. Exp Ther Med 18(4):2759–2776 31572524 10.3892/etm.2019.7886 PMC6755486

[7] Chen YH, Zhou KY, Yuan HY (2010) Research progress on the efficacy of kaempferol. Guangdong Med J 31(08):1064–1066

[8] Chen J, Zhong H, Huang Z, Chen X, You J, Zou T (2023) A Critical Review of Kaempferol in Intestinal Health and Diseases. Antioxid (Basel) 12(8):1642 10.3390/antiox12081642 10.3390/antiox12081642 PMC10451660 37627637

[9] Micek A, Godos J, Del Rio D, Galvano F, Grosso G (2021) Dietary Flavonoids and Cardiovascular Disease: A Comprehensive Dose-Response Meta-Analysis. Mol Nutr Food Res 65(6):e2001019 33559970 10.1002/mnfr.202001019

[10] Zhao W, Song D, Wang P, Tian Y, Chang S, Li W (2022) Mechanism and Experimental Verification of the Use of Rhodiola crenulata to Cytokine Storm Based on Network Pharmacology and Molecular Docking. Nat Prod Commun 17(12):1–18 10.1177/1934578X2211427

[11] Szklarczyk D, Kirsch R, Koutrouli M, Nastou K, Mehryary F, Hachilif R, Gable AL, Fang T, Doncheva NT, Pyysalo S et al (2023) The STRING database in 2023: protein-protein association networks and functional enrichment analyses for any sequenced genome of interest. Nucleic Acids Res 51(D1):D638–d646 36370105 10.1093/nar/gkac1000 PMC9825434

[12] Sherman BT, Hao M, Qiu J, Jiao X, Baseler MW, Lane HC, Imamichi T, Chang W (2022) DAVID: a web server for functional enrichment analysis and functional annotation of gene lists (2021 update). Nucleic Acids Res 50(W1):W216–w221 35325185 10.1093/nar/gkac194 PMC9252805

[13] Lanuti P, Bertagnolo V, Pierdomenico L, Bascelli A, Santavenere E, Alinari L, Capitani S, Miscia S, Marchisio M (2009) Enhancement of TRAIL cytotoxicity by AG-490 in human ALL cells is characterized by downregulation of cIAP-1 and cIAP-2 through inhibition of Jak2/Stat3. Cell Res 19(9):1079–1089 19564891 10.1038/cr.2009.80

[14] Zhu W, Ma H, Miao J, Huang G, Tong M, Zou S (2013) β-Glucan modulates the lipopolysaccharide-induced innate immune response in rat mammary epithelial cells. Int Immunopharmacol 15(2):457–465 23261364 10.1016/j.intimp.2012.12.007

[15] Jiang KF, Zhao G, Deng GZ, Wu HC, Yin NN, Chen XY, Qiu CW, Peng XL (2017) Polydatin ameliorates Staphylococcus aureus-induced mastitis in mice via inhibiting TLR2-mediated activation of the p38 MAPK/NF-κB pathway. Acta Pharmacol Sin 38(2):211–222 27890916 10.1038/aps.2016.123 PMC5309755

[16] Wu HT, Lin XX, Yang XL, Ding Y, Wang JL, Liu CL, Yu WZ (2024) Kaempferol attenuates inflammation in lipopolysaccharide-induced gallbladder epithelial cells by inhibiting the MAPK/NF-κB signaling pathway. Chem Biol Drug Des 103(4):e14519 38570708 10.1111/cbdd.14519

[17] Gholamnezhad Z, Safarian B, Esparham A, Mirzaei M, Esmaeilzadeh M, Boskabady MH (2022) The modulatory effects of exercise on lipopolysaccharide-induced lung inflammation and injury: A systemic review. Life Sci 293:120306 35016883 10.1016/j.lfs.2022.120306

[18] Shaker NS, Sahib HB, Tahseen NJ (2024) Anti-cytokine Storm Activity of Fraxin, Quercetin, and their Combination on Lipopolysaccharide-Induced Cytokine Storm in Mice: Implications in COVID-19. Iran J Med Sci 49(5):322–331 38751871 10.30476/ijms.2023.98947.3102 PMC11091274

[19] Chen XX, Min S, Shen Z, Zhang M, Huang L, Su M, Shen H, Zhu L (2025) Autophagy related biomarkers in ulcerative colitis revealed by bioinformatics analysis and immune correlation. Sci Rep 15(1):44255 41422315 10.1038/s41598-025-27363-5 PMC12722207

[20] Hu M, Ye P, Liao H, Chen M, Yang F (2016) Metformin Protects H9C2 Cardiomyocytes from High-Glucose and Hypoxia/Reoxygenation Injury via Inhibition of Reactive Oxygen Species Generation and Inflammatory Responses: Role of AMPK and JNK. *J Diabetes Res* 2016:2961954 10.1155/2016/2961954 PMC4884853 27294149

[21] Grellner W (2002) Time-dependent immunohistochemical detection of proinflammatory cytokines (IL-1beta, IL-6, TNF-alpha) in human skin wounds. Forensic Sci Int 130(2–3):90–96 12477628 10.1016/s0379-0738(02)00342-0

[22] Zhang Y, Lu Q, Hu H, Yang C, Zhao Q (2024) Esketamine alleviates hypoxia/reoxygenation injury of cardiomyocytes by regulating TRPV1 expression and inhibiting intracellular Ca(2+) concentration. Clin (Sao Paulo) 79:100363 10.1016/j.clinsp.2024.100363 PMC11070684 38692008

[23] Sun C, Li C, Hong J, Lv W, Liu Z, Wang H, Dong Q, Schiöth HB, Gao S (2025) Multi-omics profiling identifies M1 macrophage polarization-associated biomarkers in hepatitis B virus-related acute-on-chronic liver failure. Front Microbiol 16:1630042 41070131 10.3389/fmicb.2025.1630042 PMC12504312

[24] Li S, Jiang J, Yang Z, Li Z, Ma X, Li X (2018) Cardiac progenitor cell–derived exosomes promote H9C2 cell growth via Akt/mTOR activation. Int J Mol Med 42(3):1517–1525 29786755 10.3892/ijmm.2018.3699 PMC6089767

[25] Huang H-H, Li Q-F, Zhang L, Wu C-Y (2023) Clinical Efficacy of Vaccaria Segetalis Seeds and Gleditsia Sinensis Lam Thorns on Prostate Cancer: A Preliminary Mechanism Analysis Based on Network Pharmacology. Letters in Drug Design & Discovery 21(10):1874–1885 10.2174/1570180820666230502152114

[26] Lu M, Ou J, Deng X, Chen Y, Gao Q (2023) Exploring the pharmacological mechanisms of Tripterygium wilfordii against diabetic kidney disease using network pharmacology and molecular docking. Heliyon 9(6):e17550 37416640 10.1016/j.heliyon.2023.e17550 PMC10320109

[27] Ballav S, Lokhande KB, Sahu VK, Yadav RS, Swamy KV, Basu S: Identification of Novel PPAR-β/δ Agonists from Kaempferol, Quercetin, and Resveratrol Derivatives by Targeting Cancer: An Integrative Molecular Docking and Dynamics Simulation Approach. *Letters in Drug Design & Discovery* 2024, 21(4):749–762

[28] Lin X, Zhao X, Chen Q, Wang X, Wu Y, Zhao H (2023) Quercetin ameliorates ferroptosis of rat cardiomyocytes via activation of the SIRT1/p53/SLC7A11 signaling pathway to alleviate sepsis–induced cardiomyopathy. Int J Mol Med 52(6):116 10.3892/ijmm.2023.5319 10.3892/ijmm.2023.5319 PMC10635685 37859612

[29] Xue CY, Xu L, Qin QQ, Tan MT, Zong J, Li FF, Qian WH (2020) Effect and mechanism of naringenin on lipopolysaccharide-induced inflammation and apoptosis in H9c2 cardiomyocytes. Chin J Clin Pharmacol 36(22):3635–3638

[30] Mudd PA, Crawford JC, Turner JS, Souquette A, Reynolds D, Bender D, Bosanquet JP, Anand NJ, Striker DA, Martin RS et al (2020) Distinct inflammatory profiles distinguish COVID-19 from influenza with limited contributions from cytokine storm. *Sci Adv* 6(50) 10.1126/sciadv.abe3024 PMC7725462 33187979

[31] Gao M, Zhu X, Gao X, Yang H, Li H, Du Y, Gao J, Chen Z, Dong H, Wang B et al (2024) Kaempferol mitigates sepsis-induced acute lung injury by modulating the SphK1/S1P/S1PR1/MLC2 signaling pathway to restore the integrity of the pulmonary endothelial cell barrier. Chem Biol Interact 398:111085 38823539 10.1016/j.cbi.2024.111085

[32] He HL, Ren LM, Sun J (2024) Based on SIRT1/Nrf2 signaling pathway, the protective effect of kaempferol on brain injury in rats with subarachnoid hemorrhage was explored. Chin J Gerontol 44(15):3739–3743

[33] Yang H, Li D, Gao G (2024) Kaempferol Alleviates Hepatic Injury in Nonalcoholic Steatohepatitis (NASH) by Suppressing Neutrophil-Mediated NLRP3-ASC/TMS1-Caspase 3 Signaling. Molecules 29(11):2630 10.3390/molecules29112630 10.3390/molecules29112630 PMC11173805 38893506

[34] Wang F, Mei QC, Feng J, Li HJ, Wang BY, Li HZ (2024) Kaempferol regulates the ROS/TXNIP pathway on the oxidation and inflammatory injury of chondrocytes in rats with knee osteoarthritis. Chin J Gerontol 44(01):229–233

[35] Yu X, Wu Q, Ren Z, Chen B, Wang D, Yuan T, Ding H, Wang Y, Yuan G, Wang Y et al (2024) Kaempferol attenuates wear particle-induced inflammatory osteolysis via JNK and p38-MAPK signaling pathways. J Ethnopharmacol 318(Pt B):117019. 10.1016/j.jep.2023.117019 37574017

[36] Babon JJ, Kershaw NJ, Murphy JM, Varghese LN, Laktyushin A, Young SN, Lucet IS, Norton RS, Nicola NA (2012) Suppression of cytokine signaling by SOCS3: characterization of the mode of inhibition and the basis of its specificity. Immunity 36(2):239–250 22342841 10.1016/j.immuni.2011.12.015 PMC3299805

[37] Yu L, Liu Z, He W, Chen H, Lai Z, Duan Y, Cao X, Tao J, Xu C, Zhang Q et al (2020) Hydroxysafflor Yellow A Confers Neuroprotection from Focal Cerebral Ischemia by Modulating the Crosstalk Between JAK2/STAT3 and SOCS3 Signaling Pathways. Cell Mol Neurobiol 40(8):1271–1281 32060857 10.1007/s10571-020-00812-7 PMC11448784

[38] Calderón-Montaño JM, Burgos-Morón E, Pérez-Guerrero C, López-Lázaro M (2011) A review on the dietary flavonoid kaempferol. Mini Rev Med Chem 11(4):298–344 21428901 10.2174/138955711795305335

[39] Chen AY, Chen YC (2013) A review of the dietary flavonoid, kaempferol on human health and cancer chemoprevention. Food Chem 138(4):2099–2107 23497863 10.1016/j.foodchem.2012.11.139 PMC3601579

[40] Maeda N, Hashimoto A, Morita R, Munemasa S, Murata Y, Nakamura Y, Nakamura T (2026) Comparison of bioavailability of quercetin and its structural analogs in mice. Arch Biochem Biophys 779:110775 41724417 10.1016/j.abb.2026.110775

[41] Chu T, Wang Y, Wang S, Li J, Li Z, Wei Z, Li J, Bian Y (2025) Kaempferol regulating macrophage foaming and atherosclerosis through Piezo1-mediated MAPK/NF-κB and Nrf2/HO-1 signaling pathway. J Adv Res 75:635–650 39561922 10.1016/j.jare.2024.11.016 PMC12789723

[42] Li X, Hu X, Yang F, Hu G, Yuan L, Li J (2025) The Effect of Kaempferol on Valsartan Metabolism In Vitro and In Vivo and the Underlying Mechanism With Cytochrome p450 Using UPLC-MS/MS. Biomed Chromatogr 39(9):e70184 40771041 10.1002/bmc.70184

[43] Akiyama M, Mizokami T, Ito H, Ikeda Y (2023) A randomized, placebo-controlled trial evaluating the safety of excessive administration of kaempferol aglycone. Food Sci Nutr 11(9):5427–5437 37701215 10.1002/fsn3.3499 PMC10494647

[44] Kim GD (2017) Kaempferol Inhibits Angiogenesis by Suppressing HIF-1α and VEGFR2 Activation via ERK/p38 MAPK and PI3K/Akt/mTOR Signaling Pathways in Endothelial Cells. Prev Nutr Food Sci 22(4):320–326 29333385 10.3746/pnf.2017.22.4.320 PMC5758096

[45] Zhu F, Cheng H, Luo Z, Cao A, Yu T, Ge H, Hu S, Xie Z, Li D (2025) Theogallin Protects Myocardial Ischemia-Reperfusion Injury by Inhibiting the Interleukin-17 Signaling Pathway. J Agric Food Chem 73(31):19373–19385 40690952 10.1021/acs.jafc.4c10675 PMC12333330

[46] Yang Q, Ji H, Modarresi Chahardehi A (2025) JAK/STAT pathway in myocardial infarction: Crossroads of immune signaling and cardiac remodeling. Mol Immunol 186:206–217 40882407 10.1016/j.molimm.2025.08.018

[47] Liu JP, Shen KY, Cheng WC, Chang WC, Hsieh CY, Lo CC, Kuo TT, Lin CC, Liu SJ, Huang WC et al (2024) ADAM9 drives the immunosuppressive microenvironment by cholesterol biosynthesis-mediated activation of IL6-STAT3 signaling for lung tumor progression. Am J Cancer Res 14(4):1850–1865 38726266 10.62347/LODV2387 PMC11076253

[48] Liu A, Zhao F, Wang J, Zhao Y, Luo Z, Gao Y, Shi J (2016) Regulation of TRPM7 Function by IL-6 through the JAK2-STAT3 Signaling Pathway. PLoS ONE 11(3):e0152120 27010689 10.1371/journal.pone.0152120 PMC4806911

[49] Billing U, Jetka T, Nortmann L, Wundrack N, Komorowski M, Waldherr S, Schaper F, Dittrich A (2019) Robustness and Information Transfer within IL-6-induced JAK/STAT Signalling. Commun Biol 2:27 30675525 10.1038/s42003-018-0259-4 PMC6338669

[50] Chen Y, Luo Y, Liu Y, Luo D, Liu A (2025) Dual efficacy of tocilizumab in managing PD-1 inhibitors-induced myocardial inflammatory injury and suppressing tumor growth with PD-1 inhibitors: a preclinical study. Cancer Immunol Immunother 74(2):52 39752010 10.1007/s00262-024-03899-9 PMC11699076

[51] Nie J, Zhou L, Tian W, Liu X, Yang L, Yang X, Zhang Y, Wei S, Wang DW, Wei J (2025) Deep insight into cytokine storm: from pathogenesis to treatment. Signal Transduct Target Ther 10(1):112 40234407 10.1038/s41392-025-02178-y PMC12000524

[52] Wu X, Li X, Yang C, Diao Y (2021) Target Characterization of Kaempferol against Myocardial Infarction Using Novel In Silico Docking and DARTS Prediction Strategy. Int J Mol Sci 22(23):12908 10.3390/ijms222312908 10.3390/ijms222312908 PMC8657499 34884711

[53] van der Pol A, van Gilst WH, Voors AA, van der Meer P (2019) Treating oxidative stress in heart failure: past, present and future. Eur J Heart Fail 21(4):425–435 30338885 10.1002/ejhf.1320 PMC6607515

[54] Zhang XE, Pang YB, Bo Q, Hu SY, Xiang JY, Yang ZR, Zhang XM, Chen AJ, Zeng JH, Ma X et al (2023) Protective effect of paeoniflorin in diabetic nephropathy: A preclinical systematic review revealing the mechanism of action. PLoS ONE 18(9):e0282275 37733659 10.1371/journal.pone.0282275 PMC10513216

