# Supplementary figures and images for: Kaempferol attenuates LPS-induced inflammatory responses in H9c2 cells through involvement of the IL-6/JAK2/STAT3 pathway
Source: Mol Biol Rep. 2026 Apr 27;53(1):673. doi: 10.1007/s11033-026-11855-2 (PMC13121200; doi:10.1007/s11033-026-11855-2)

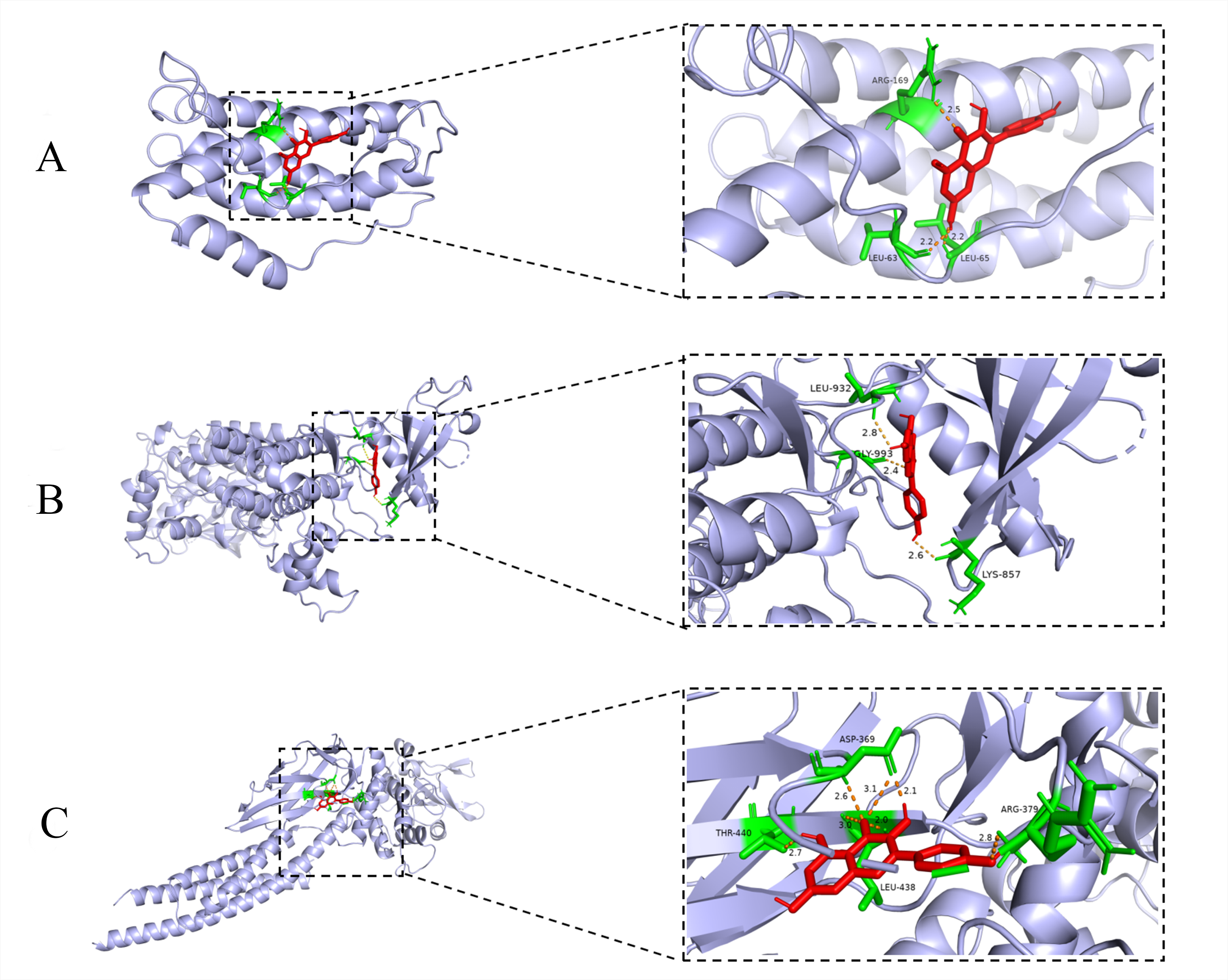

Supplement: Supplementary file 2 — Supplementary Material 2 [file 11033_2026_11855_MOESM2_ESM.tif]

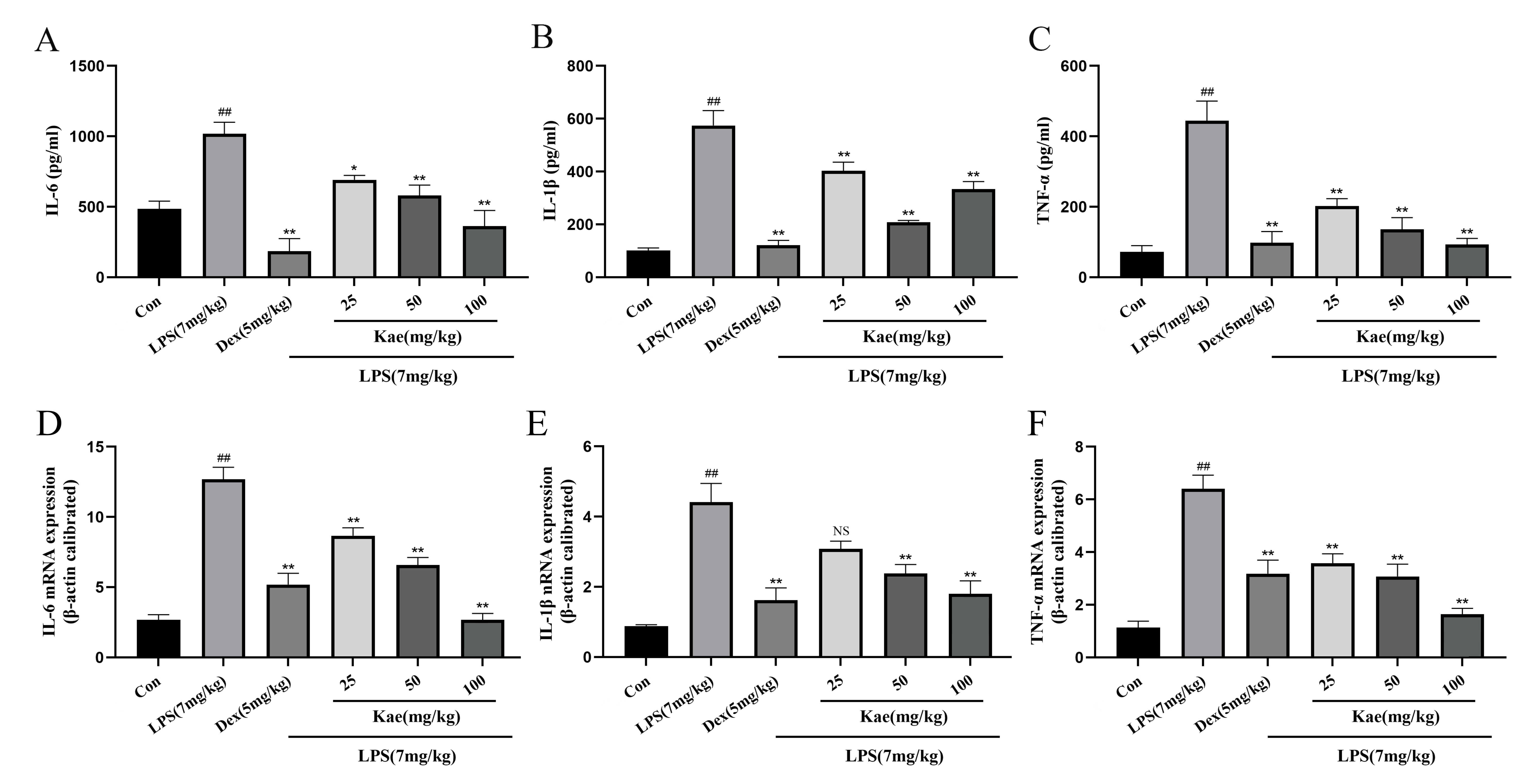

Supplement: Supplementary file 3 — Supplementary Material 3 [file 11033_2026_11855_MOESM3_ESM.tif]

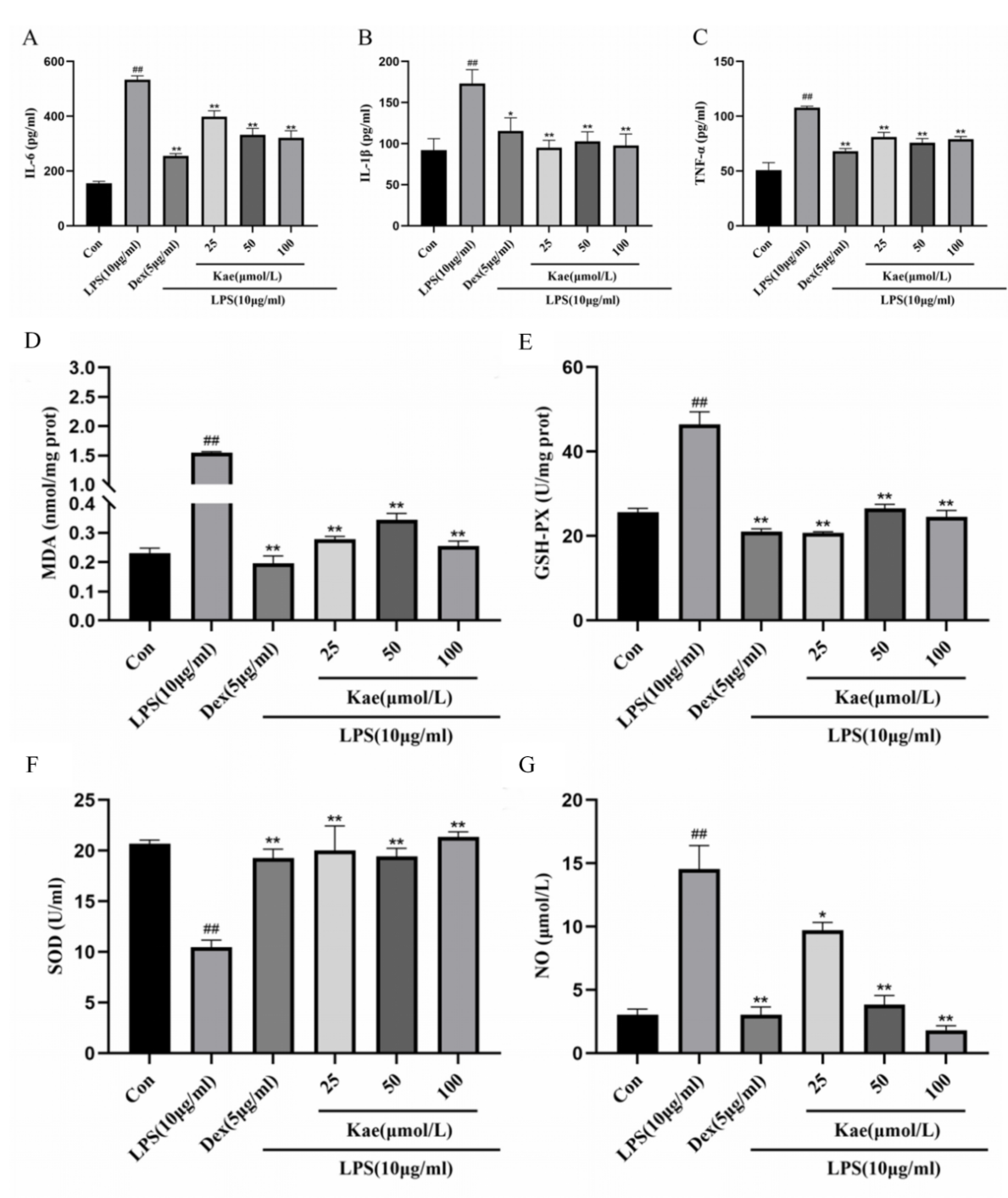

Supplement: Supplementary file 4 — Supplementary Material 4 [file 11033_2026_11855_MOESM4_ESM.tif]
