# Supplementary material for: Kaempferol attenuates LPS-induced inflammatory responses in H9c2 cells through involvement of the IL-6/JAK2/STAT3 pathway
Source: Mol Biol Rep. 2026 Apr 27;53(1):673. doi: 10.1007/s11033-026-11855-2 (PMC13121200; doi:10.1007/s11033-026-11855-2)
Supplement: Supplementary file 5 — Supplementary Material 5 [file 11033_2026_11855_MOESM5_ESM.docx]

**Supplementary Table 1.** Anti-inflammatory effects and mechanisms of Kae in related diseases.

| **Subjects and Disease Models** | **Signaling Pathways Involved** | **Mechanism of Action** | **Primary Inhibited Cytokines** |
| --- | --- | --- | --- |
| Mice and Acute Lung Injury | SphK1/S1P/S1PR1/MLC2 Signaling Pathway | Kae inhibits hyperphosphorylation of MLC2 and decreases the levels of SphK1 and S1PR1, thereby strengthening the endothelial barrier and reducing the inflammatory response. | IL-6, IL-1β, and TNF-α |
| Rats and Subarachnoid Hemorrhage | SIRT1/Nrf2 Signaling Pathway | Kae can reduce inflammation and oxidative stress in rats with subarachnoid hemorrhage, while also enhancing neurological function and minimizing neuronal damage. | IL-6, TNF-α, MDA, and SOD |
| Mice and Nonalcoholic Steatohepatitis | NLRP3-ASC/TMS1-Caspase 3 Signaling Pathway | Kae can reduce lipid droplet accumulation and downregulate the expression of NLRP3-ASC/TMS1-Caspase 3, thereby targeting inflammation and fat metabolism. | IL-6, IL-1β, MCP-1, and TNF-α |
| Rats and Knee Osteoarthritis | ROS/TXNIP Signaling Pathway | Kae can inhibit the ROS/TXNIP pathway, thereby reducing oxidative stress and inflammatory damage in rat chondrocytes affected by knee osteoarthritis. | IL-6, TNF-α, IL-1β, MDA, NO, GSH-Px, and NLRP3 |
| Mice and Inflammatory Osteolysis | JNK and p38 MAPK Signaling Pathway | Kae downregulates the conduction of JNK and p38/MAPK pathways, as well as the expression of NFATc1, thereby reducing inflammation and exerting anti-osteoclast effects. | IL-6, IL-1β, and TNF-α |

Note: SphK1, Sphingosine kinase 1 ; S1P, Sphingosine-1-phosphate; S1PR1, S1P receptor 1; MLC2, Myosin light chain 2; PI3K, Phosphoinositide 3-kinase; AKT; Threonine kinase 1; mTOR, Mammalian target of rapamycin; SIRT1, Silent information regulator 1; Nrf2, Nuclear factor erythroid 2-realated factor 2; ROS, Reactive oxygen species; TXNIP, Thioredoxin interacting protein; JNK, Jun kinase; p38-MAPK, p38 mitogen-activated protein kinase.
