# Supplementary material for: Kaempferol attenuates LPS-induced inflammatory responses in H9c2 cells through involvement of the IL-6/JAK2/STAT3 pathway
Source: Mol Biol Rep. 2026 Apr 27;53(1):673. doi: 10.1007/s11033-026-11855-2 (PMC13121200; doi:10.1007/s11033-026-11855-2)

JAK2-1


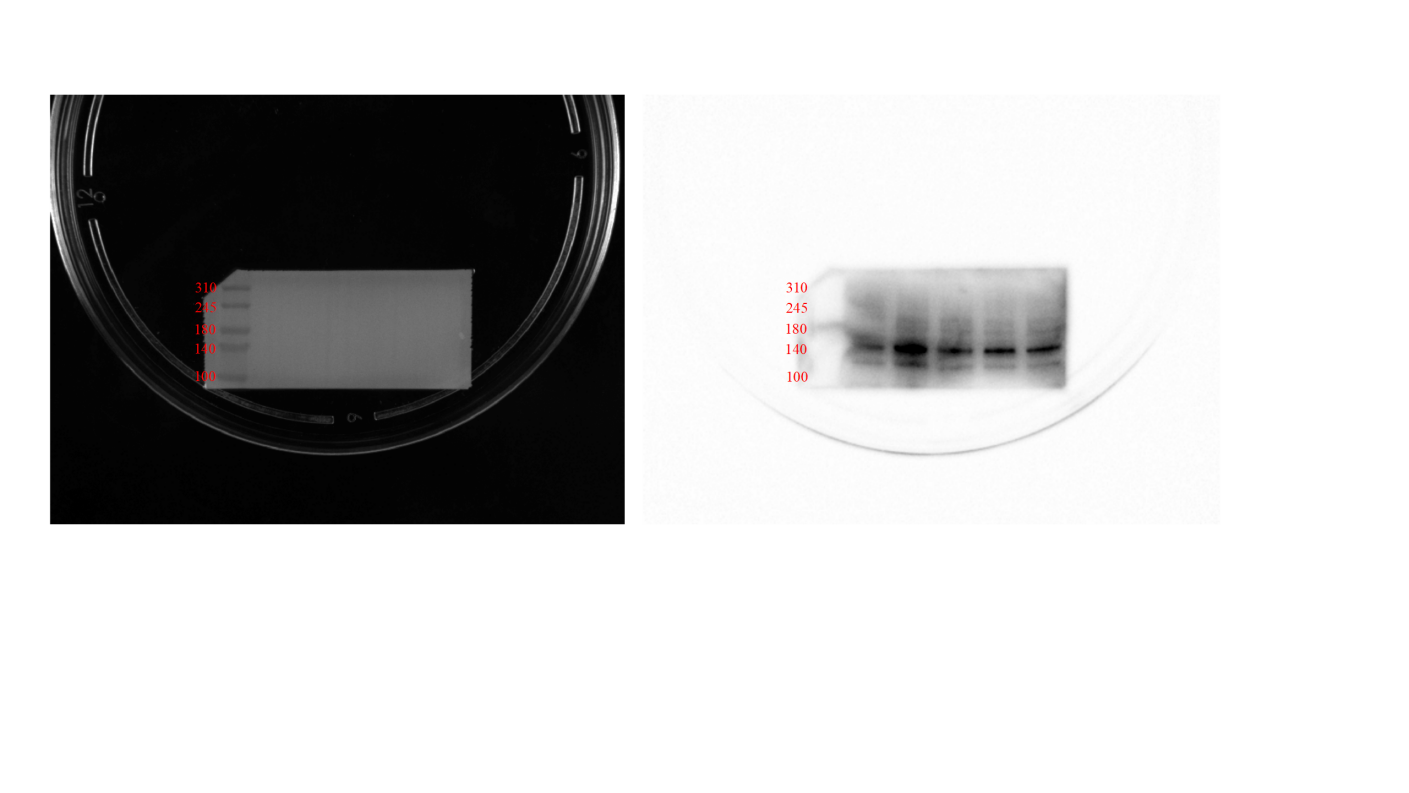


JAK2-2


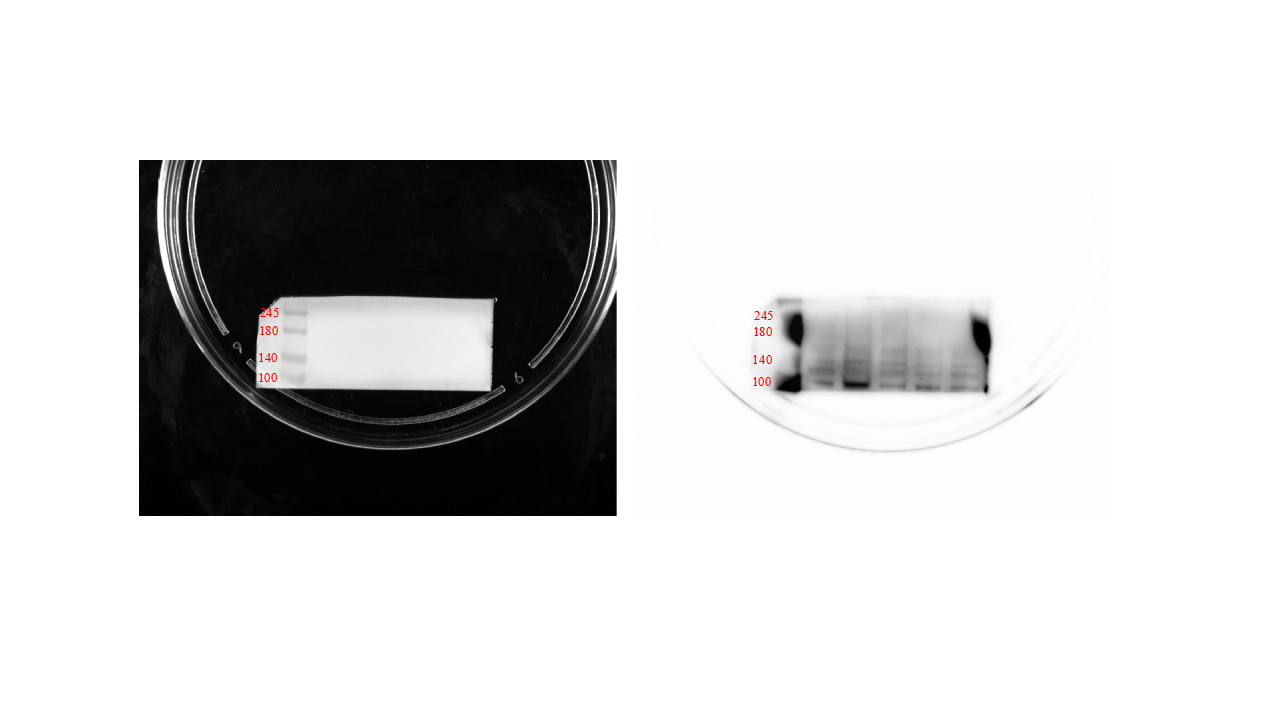


JAK2-3


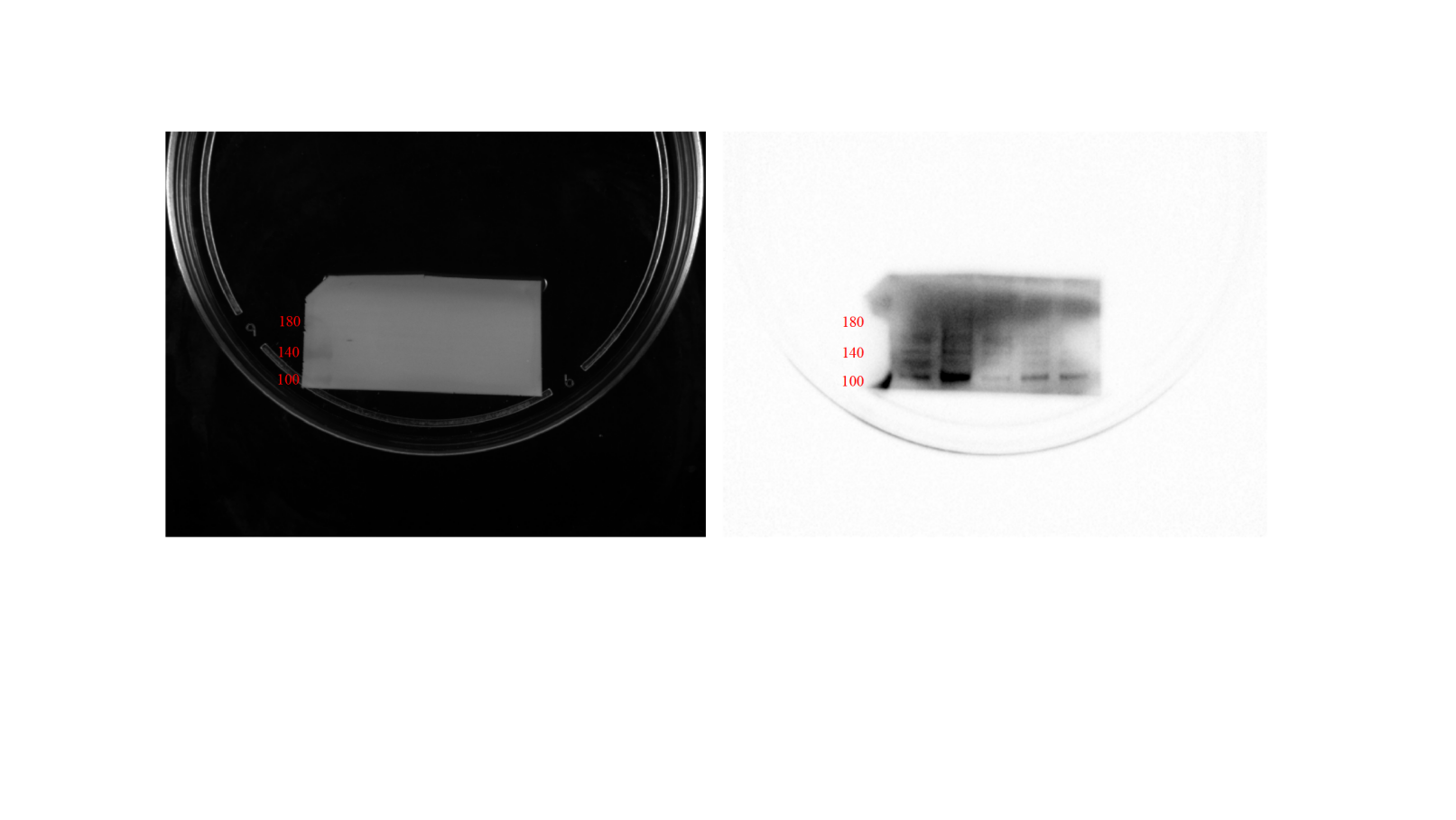


STAT3-1


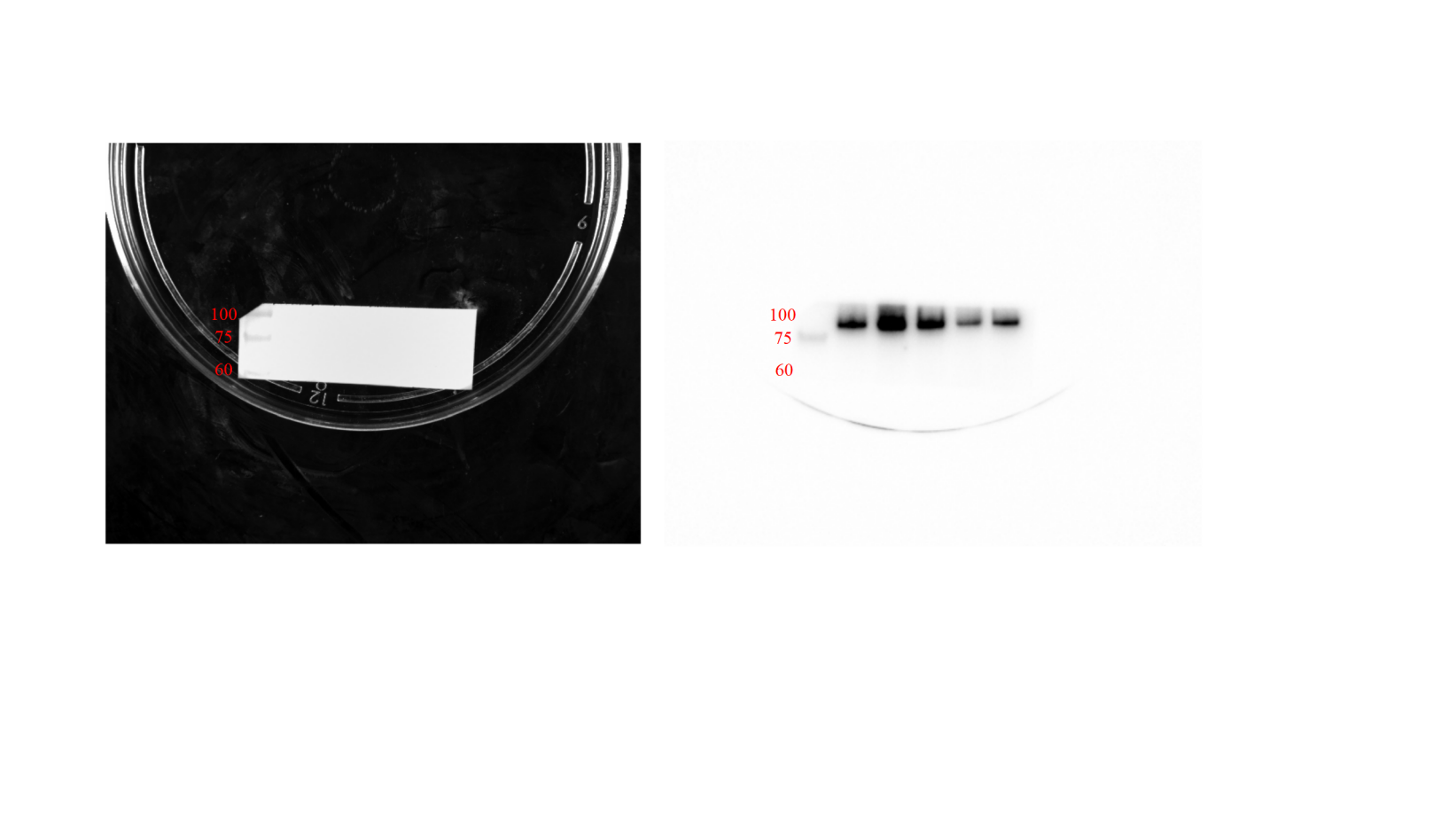


STAT3-2


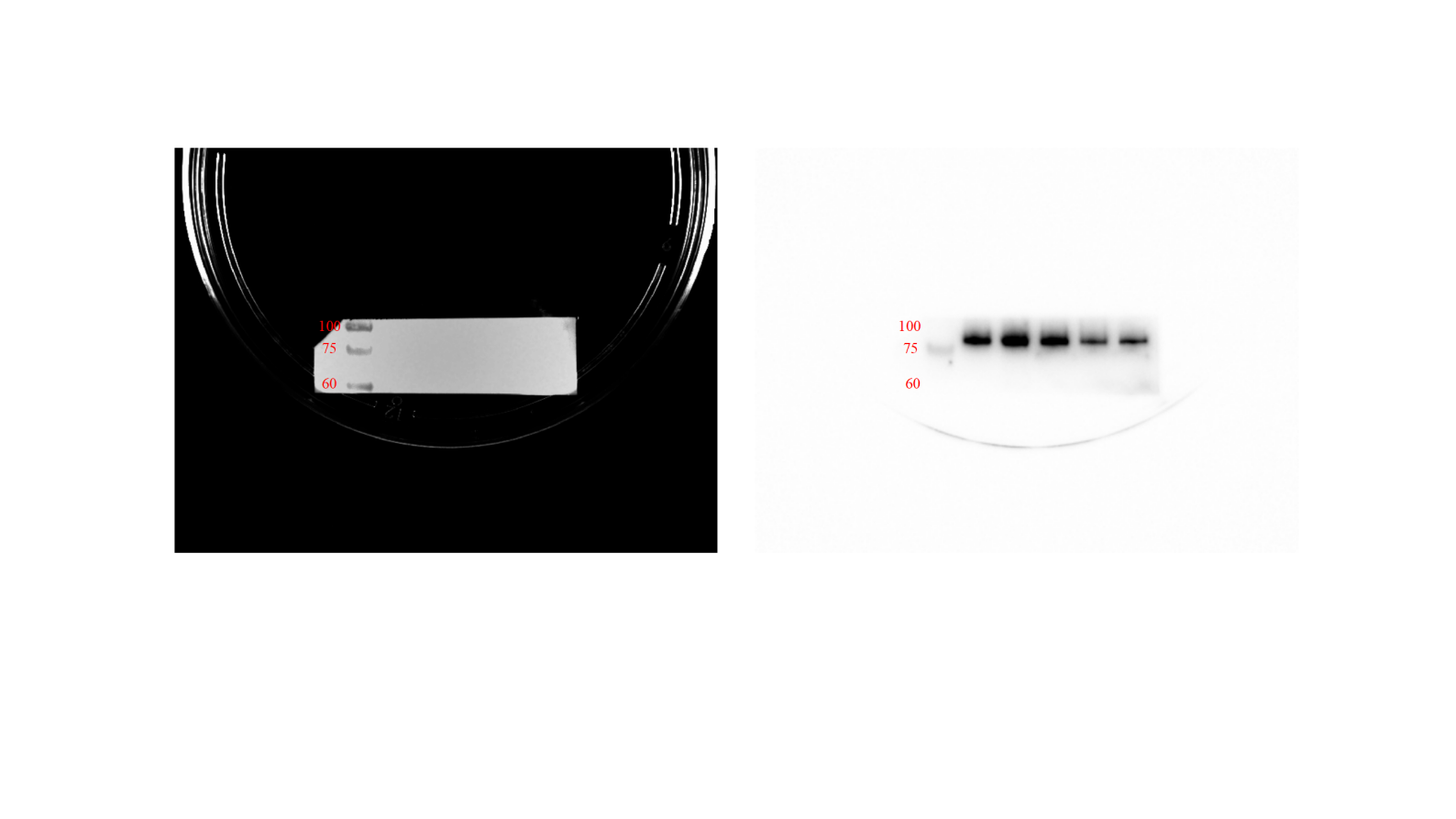


STAT3-3


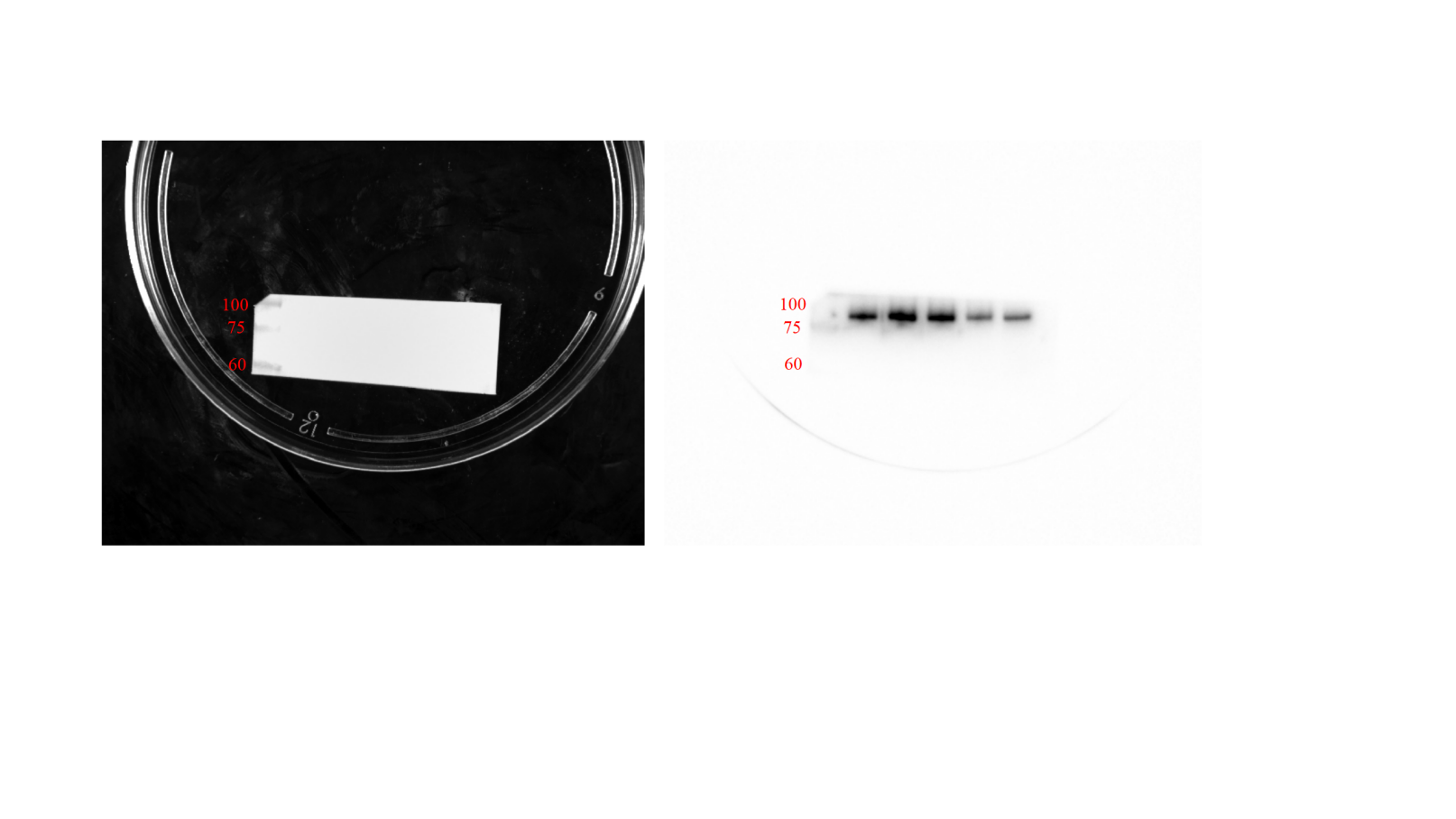


β-actin-1
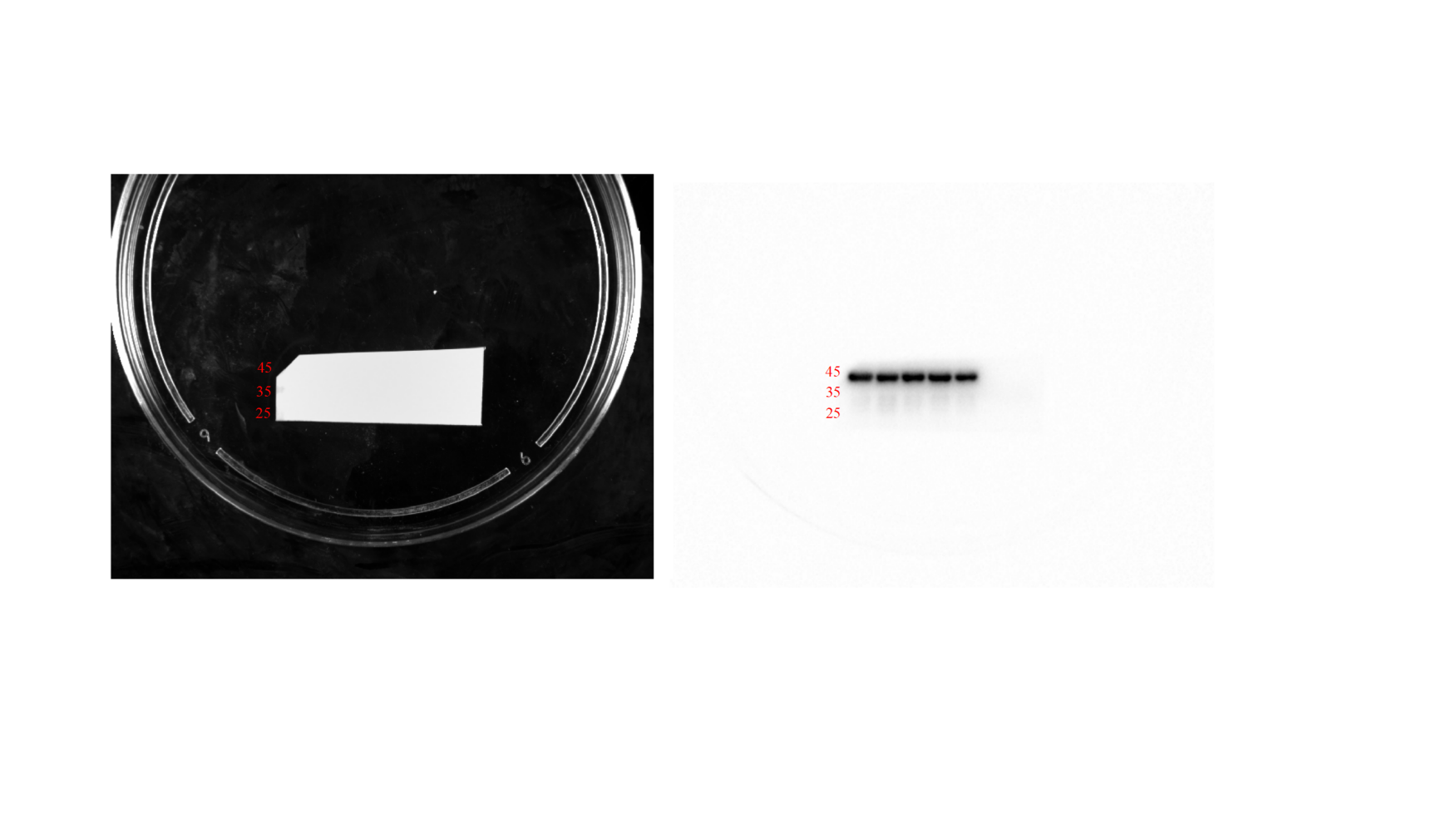


β-actin-2


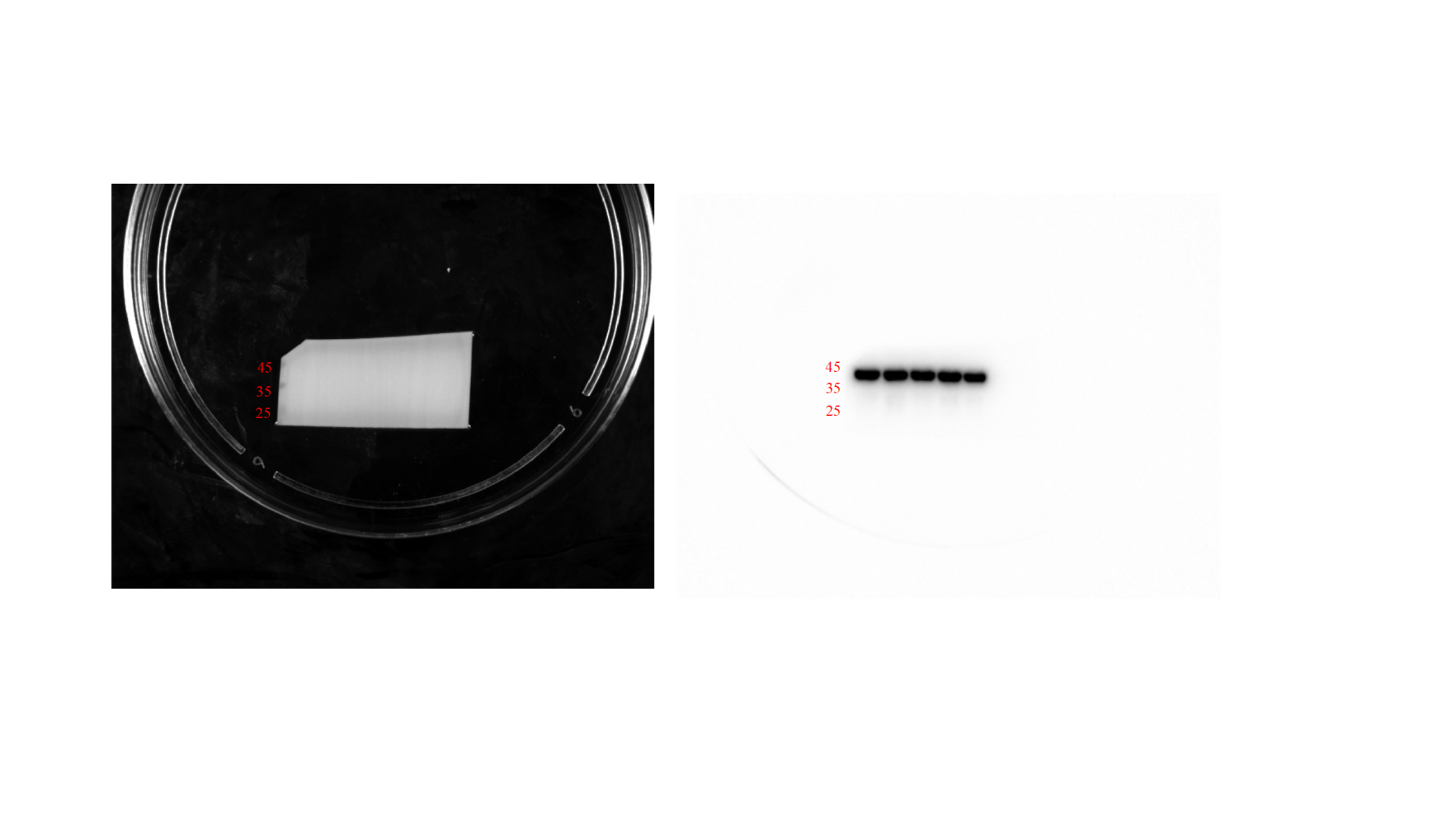


β-actin-3


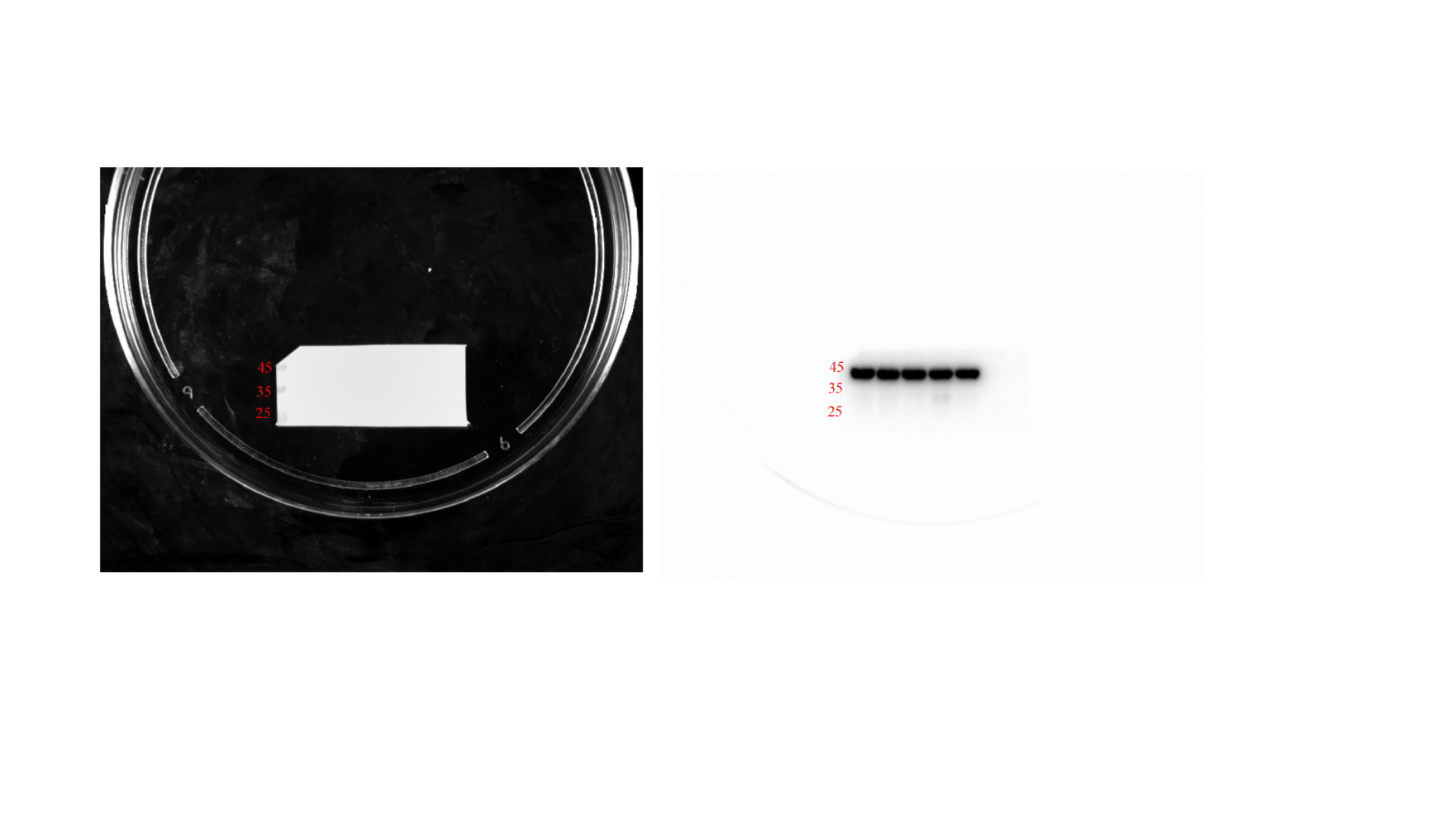


P-JAK2-1


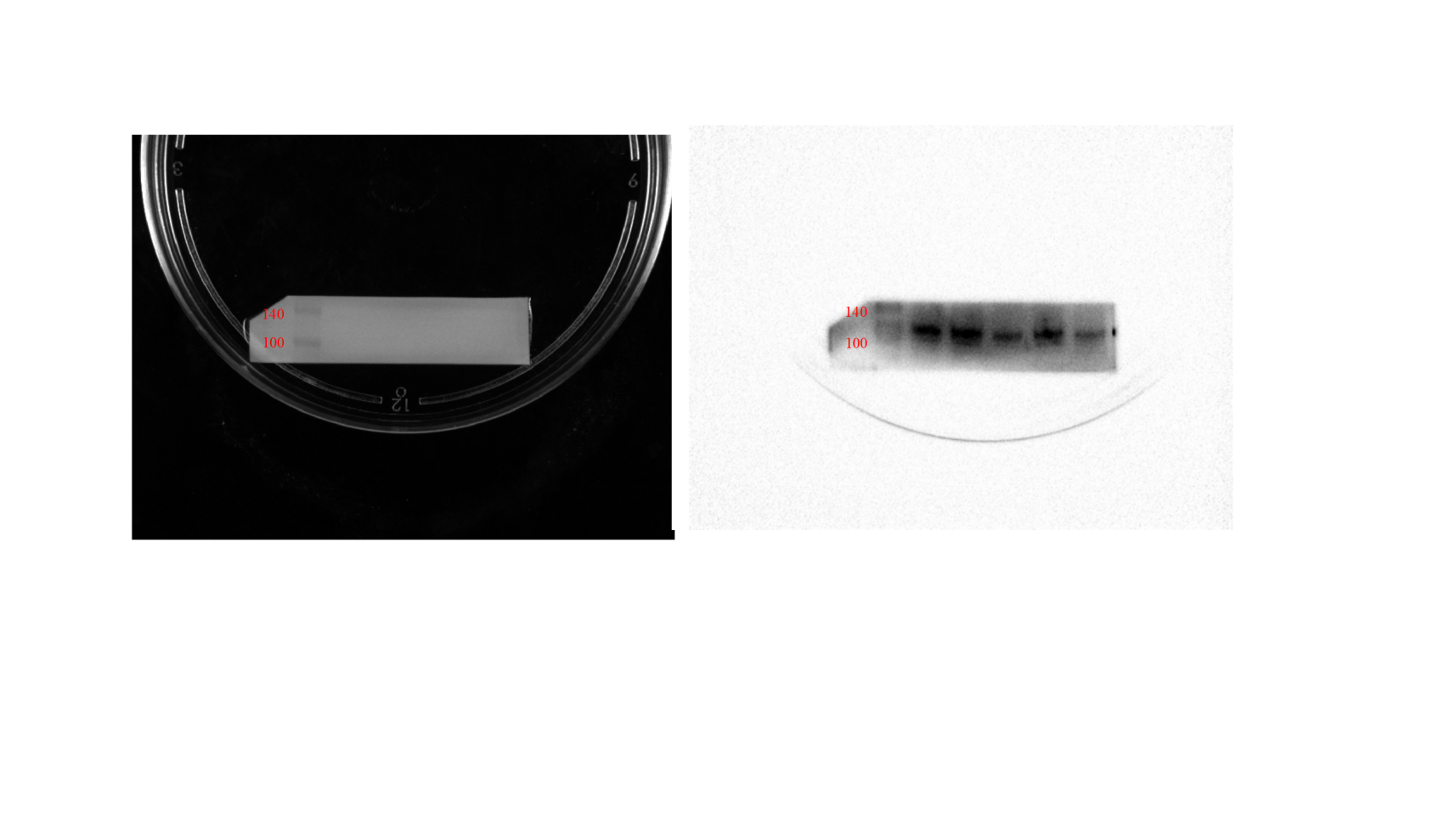


P-JAK2-2


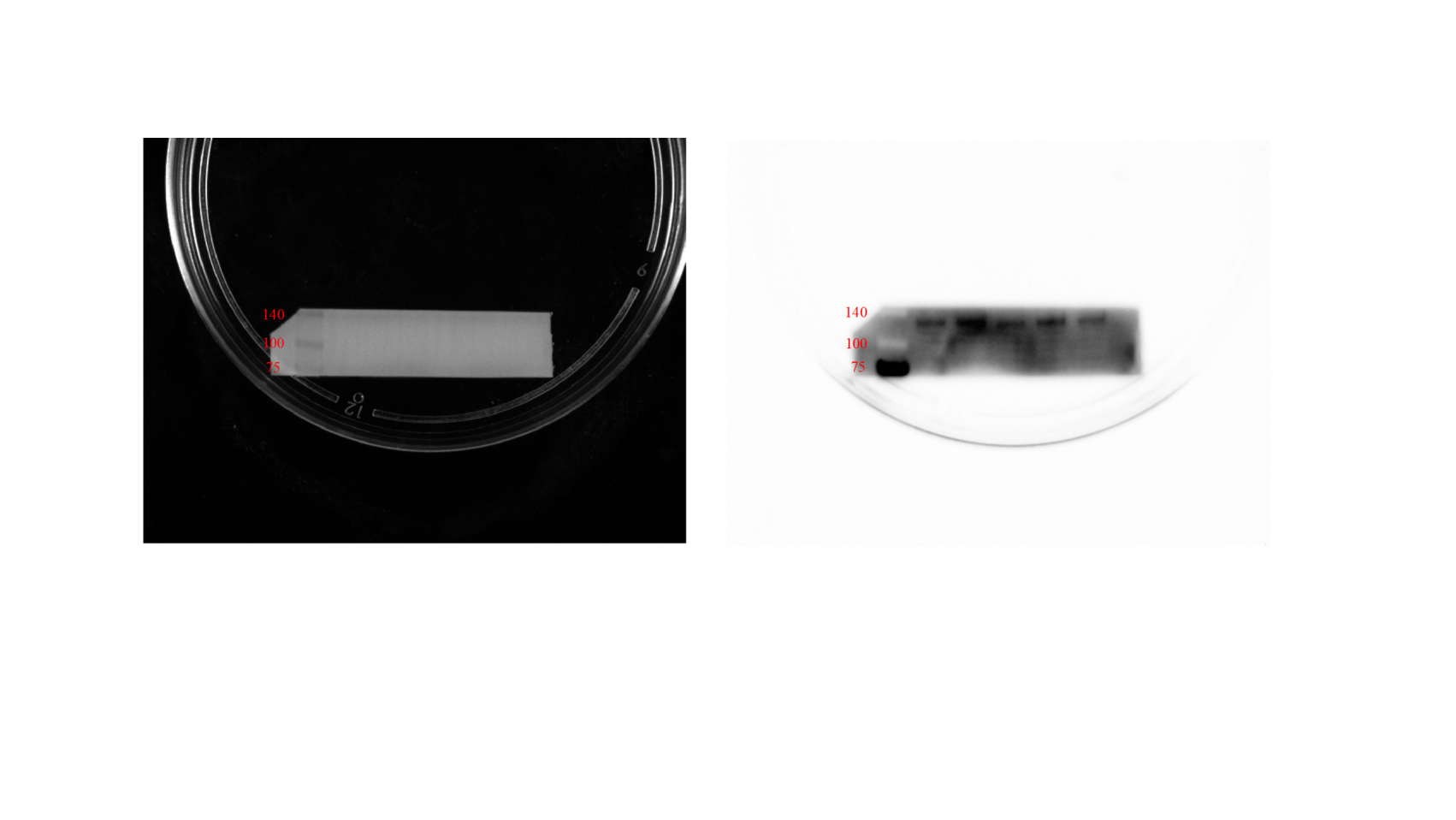


P-JAK2-3


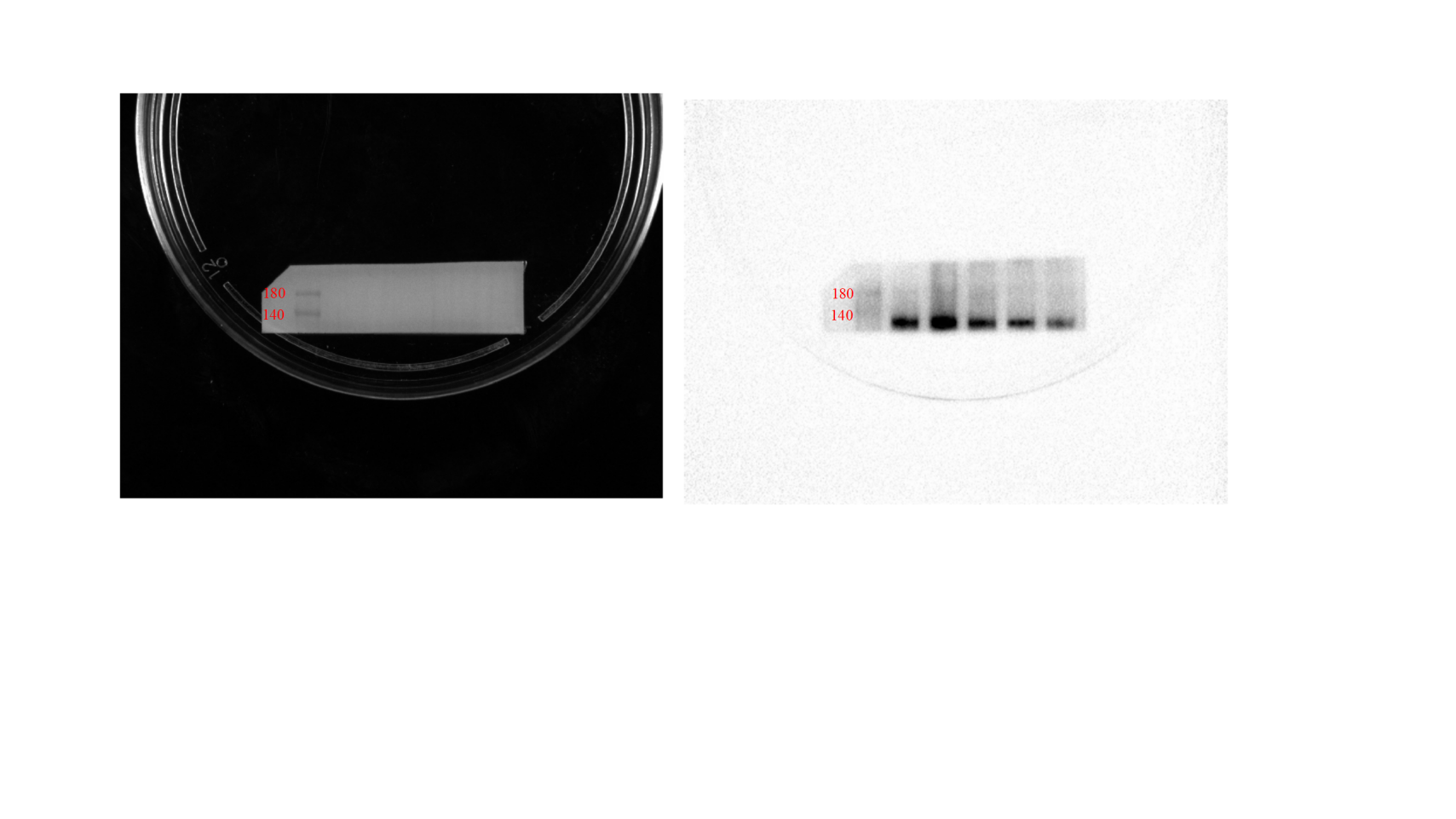


P-STAT3-1


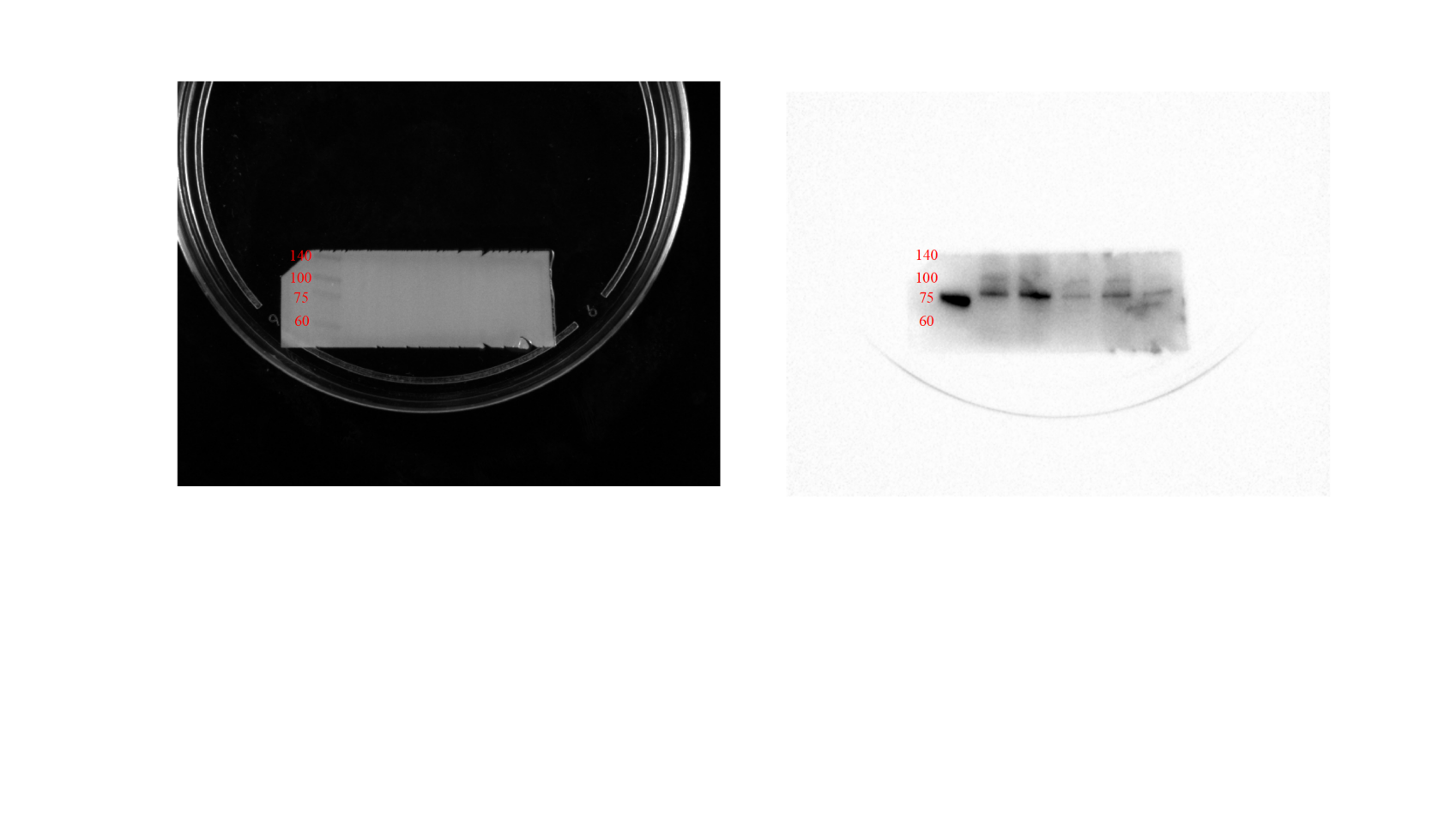


P-STAT3-2


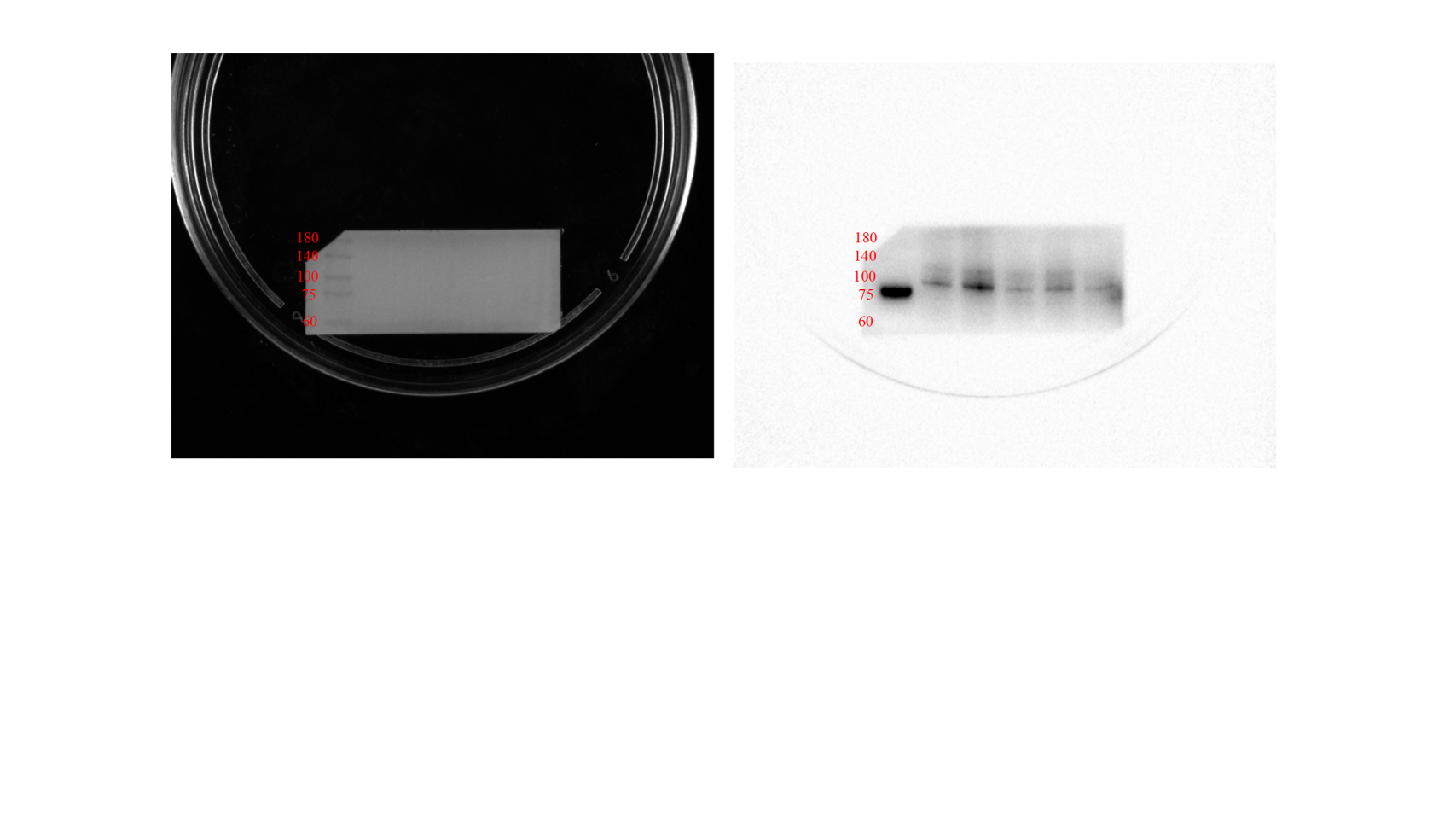


P-STAT3-3


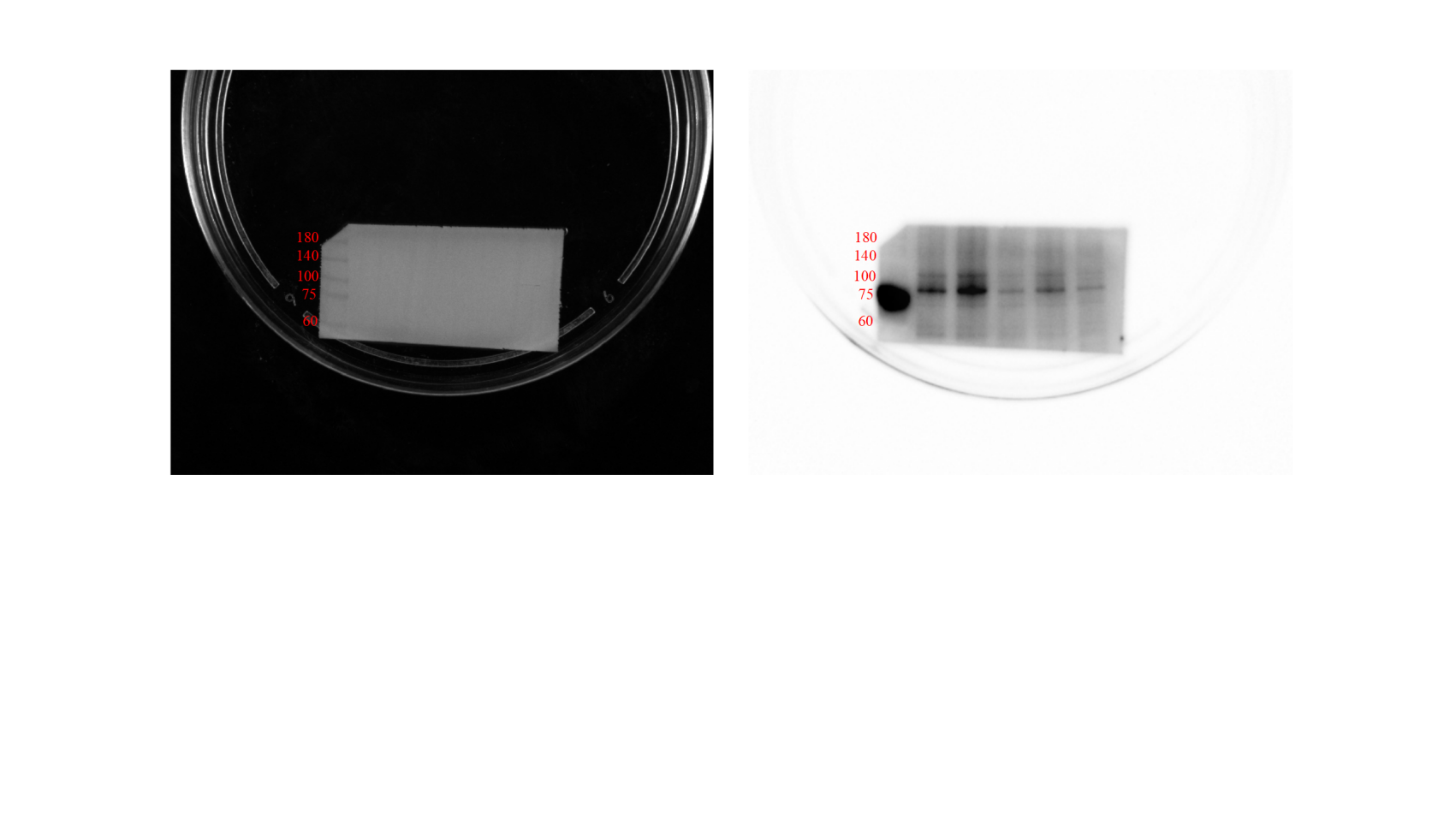


β-actin-1


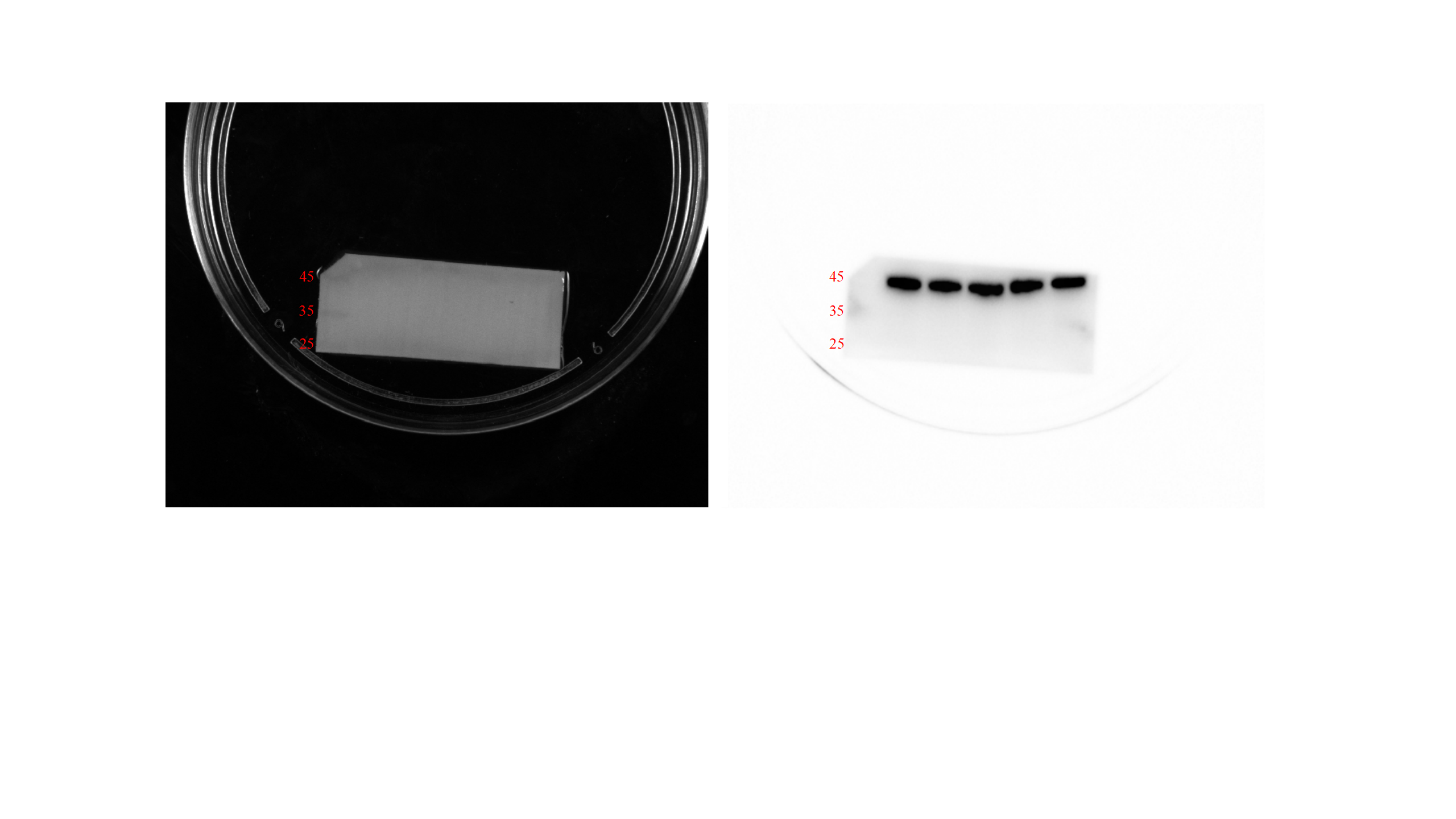


β-actin-2


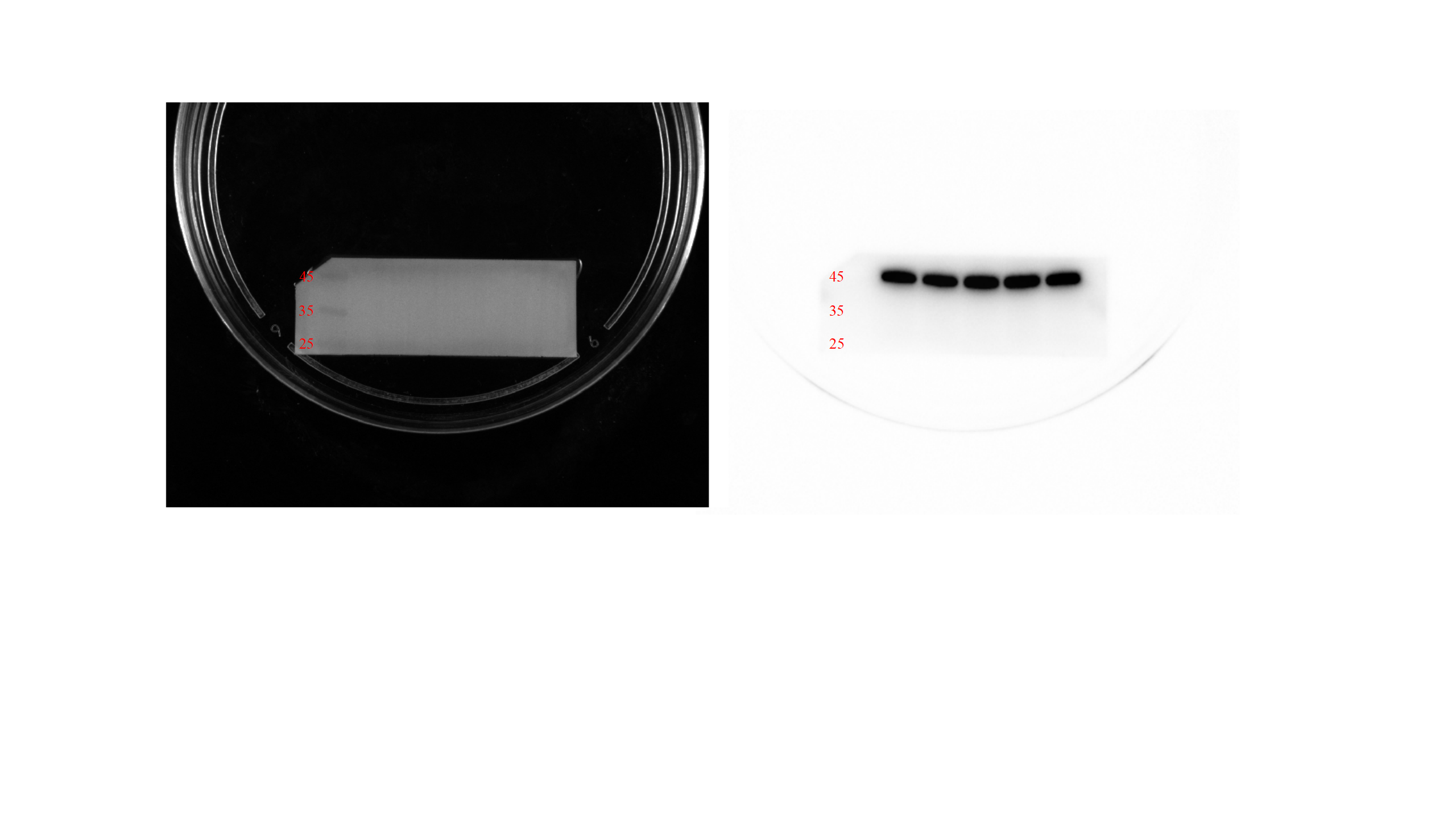


β-actin-3


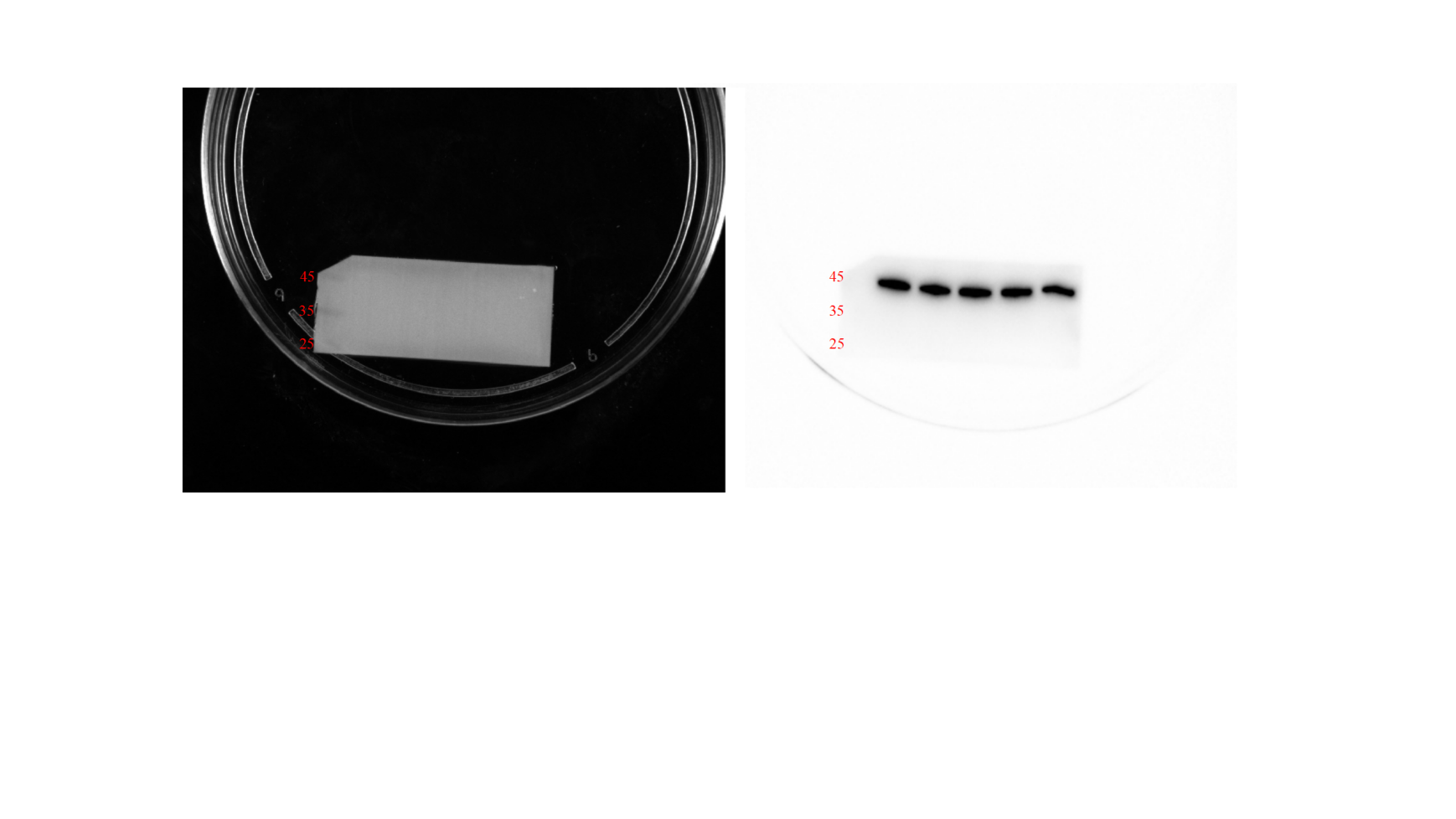

Supplement: Supplementary file 6 — Supplementary Material 6 [file 11033_2026_11855_MOESM6_ESM.docx]
